# Single-cell transcriptomics reveals intestinal cell heterogeneity and identifies *Ep300* as a potential therapeutic target in mice with acute liver failure

**DOI:** 10.1038/s41421-023-00578-4

**Published:** 2023-07-25

**Authors:** Jie Yin, Ziming Zhao, Jianzheng Huang, Yang Xiao, Mewlude Rehmutulla, Biqiong Zhang, Zijun Zhang, Ming Xiang, Qingyi Tong, Yonghui Zhang

**Affiliations:** Hubei Key Laboratory of Natural Medicinal Chemistry and Resource Evaluation, School of Pharmacy, Tongji Medical College, Huazhong University of Science and Technology, Wuhan, Hubei China

**Keywords:** Bioinformatics, Mechanisms of disease

## Abstract

Acute liver failure (ALF) is a severe life-threatening disease associated with the disorder of the gut-liver axis. However, the cellular characteristics of ALF in the gut and related therapeutic targets remain unexplored. Here, we utilized the D-GALN/LPS (D/L)-induced ALF model to characterize 33,216 single-cell transcriptomes and define a mouse ALF intestinal cellular atlas. We found that unique, previously uncharacterized intestinal immune cells, including T cells, B cells, macrophages, and neutrophils, are responsive to ALF, and we identified the transcriptional profiles of these subsets during ALF. We also delineated the heterogeneity of intestinal epithelial cells (IECs) and found that ALF-induced cell cycle arrest in intestinal stem cells and activated specific enterocyte and goblet cell clusters. Notably, the most significantly altered IECs, including enterocytes, intestinal stem cells and goblet cells, had similar activation patterns closely associated with inflammation from intestinal immune activation. Furthermore, our results unveiled a common *Ep300*-dependent transcriptional program that coordinates IEC activation during ALF, which was confirmed to be universal in different ALF models. Pharmacological inhibition of *Ep300* with an inhibitor (SGC-CBP30) inhibited this cell-specific program, confirming that *Ep300* is an effective target for alleviating ALF. Mechanistically, *Ep300* inhibition restrained inflammation and oxidative stress in the dysregulated cluster of IECs through the P38-JNK pathway and corrected intestinal ecology by regulating intestinal microbial composition and metabolism, thereby protecting IECs and attenuating ALF. These findings confirm that *Ep300* is a novel therapeutic target in ALF and pave the way for future pathophysiological studies on ALF.

## Introduction

Acute liver failure (ALF), a severe liver dysfunction syndrome with a high fatality rate, is caused mainly by drug abuse, liver toxins, hepatitis virus, autoimmunity and ischemia. It is characterized by rapid deterioration of liver function and occurs in patients who do not have preexisting liver disease, leading to mental state changes, coagulation dysfunction (usually an international normalized ratio ≥1.5), multiorgan failure and mortality^1^. The main therapeutic options for ALF are supportive treatment and liver transplantation, both of which are limited by high cost and uncertain effects. In addition, the scarcity of donors severely limits the application of liver transplantation^1,2^, highlighting the imperative for fundamental research into ALF and the search for new relevant therapeutic targets for drug development to improve the treatment of ALF.

The gut–liver axis refers to the bidirectional crosstalk between the gut and the liver. An imbalance in gut homeostasis results in a breach of the gut barrier. After that, microbial antigens, metabolites and other substances can reach the liver through the portal circulation and affect hepatic immunity and inflammation; meanwhile, the immune cells in the gut can also reach the liver through the lymphatic circulation^3^. Thus, the gut–liver axis plays a critical role in the body and controls the gastrointestinal tract and liver health. Advances in the gut–liver axis research have provided important insights into the pathophysiology and potential therapeutic targets of several liver diseases^4^, including ALF^5^. These advances have encouraged more research on the gut and its microbiome to improve the understanding and treatment of ALF. However, comprehensive, high-resolution cellular characterization of the events in the intestine leading to liver insufficiency in ALF has not been conducted, and the related therapeutic targets remain unexplored.

The intestinal mucosa, which possesses a vast surface area, plays a crucial role as an immune barrier against potential pathogens. It functions as a site of interaction between foreign substances and those present in the gut and liver, allowing nutrients to enter the circulation and reach the liver while limiting the systemic dissemination of microbes and toxins^6^. The mucosal immune barrier is a selectively permeable barrier that consists of physical, immune, and microbial components, and intestinal epithelial cells (IECs) are key regulators of the physical barrier^7^. Microbial signals can be recognized by IECs and further affect intestinal homeostasis and barrier function^8^. In addition to IECs, immune cells also participate in the maintenance of intestinal homeostasis. Peyer’s patches (PPs), located mainly in the ileum, are organized interlymphatic tissues of the intestine and are key routes for the invasion of many pathogens into the intestine^9,10^. PPs play an important role in intestinal mucosal immunity by recognizing intestinal luminal antigens through epithelial microfold cells (M cells) and delivering them to B cells via the mononuclear phagocytic system, thereby triggering a specific immune response^11^. In summary, the intestinal epithelium (IE) and PPs represent the main tissues of the intestine, and they play a substantial role in shaping the preimmune repertoire in the intestine and tailoring mucosal immune responses.

In the present study, using single-cell RNA sequencing (scRNA-seq), a state-of-the-art technology for unraveling the heterogeneity and complexity of individual cells within highly organized tissues^12^, we performed a comprehensive and comparative phenotypic analysis of the intestinal cellular atlas in a D-galactosamine/lipopolysaccharide (D-GALN/LPS, D/L)-induced mouse ALF model. We further identified which kinds of immune cell subsets were activated and how the IECs clusters changed in the ALF mouse model. *Ep300* (E1A binding protein p300) is a key transcriptional coactivator and histone acetyltransferase that is crucial to key cellular processes such as cell differentiation, proliferation, and hypoxia response; it is also an attractive target for many diseases^13,14^. Herein, we identified and confirmed that *Ep300* is an effective target for ALF treatment, and explored the mechanism of action of *Ep300* inhibition. Our findings demonstrate, for the first time, the intestinal cellular landscape of ALF and provide valuable insights into the protective effects of *Ep300* inhibition in ALF.

## Results

### Intestinal cell heterogeneity in mice with ALF

**Fig. 1:**
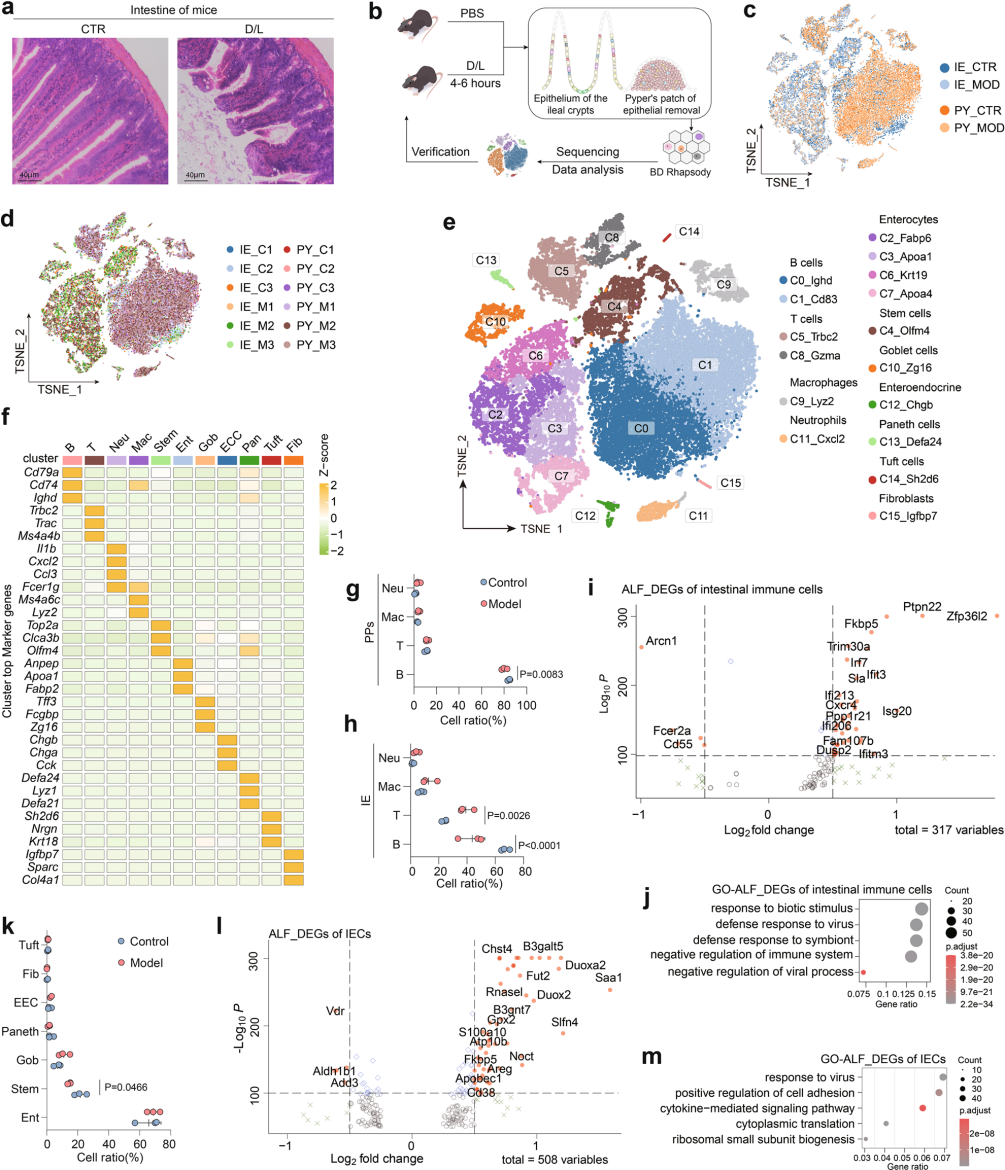
Intestinal cell heterogeneity in D/L-induced ALF mice. **a** Representative H&E-stained mouse ileal sections. Scale bars, 40 μm. **b** Schematic overview of the scRNA-seq experiment. **c**, **d** UMAP plots of all cells clustered and color-coded by four groups or 12 mice. **e** Major cell clusters and respective cell-type assignments in UMAP. **f** Heatmap of marker gene expression in each cell cluster (top three marker genes). **g**, **h** Percentages of immune cells in PPs or IE. Means ± SD, two-way ANOVA, *n* = 6 mice for IE or PPs. **i** Volcano plot of ALF_DEGs of immune cells. **j** Top five terms in the GO term enrichment analysis for genes upregulated in immune cells of ALF mice compared to control mice. **k** Percentages of each type of IECs in control (*n* = 3) and model (*n* = 3) mice. Means ± SD, two-way ANOVA. **l** Volcano plot of ALF_DEGs of IECs. **m** Top five terms in the GO term enrichment analysis for genes upregulated in IECs of ALF mice compared to control mice.

In the classic D/L-induced ALF mouse model, we observed the breakage of intestinal villi, extensive exposure of intestinal crypts, and disruption of intestinal tight junctions (Fig. 1a). To further investigate these changes, we collected IE and PPs without IE^15^ (colored tissue in Fig. 1b) from D/L-induced ALF model mice for scRNA-seq analysis based on microtiter plates. After quality control, 33,216 high-quality cells obtained from 12 mice were analyzed (Fig. 1c, d). Unsupervised clustering partitioned the cells into 16 clusters (C0 to C15), which were visualized using t-distributed stochastic neighbor embedding (tSNE)^16^ (Fig. 1e). Eleven major cell types were identified by accepted expression signatures, as follows: B cells (B, *Cd79a*^+^), T cells (T, *Trac*^+^), macrophages (Mac, *Lyz2*^+^), neutrophils (Neu, *Cxcl2*^+^), enterocytes (Ent, *Fabp2*^+^), stem cells (Stem, *Olfm*4^+^), goblet cells (Gob, *Tff3*^+^), enteroendocrine cells (EEC, *Chgb*^+^), Paneth cells (Pan, *Defa24*^+^), tuft cells (Tuft, *Sh2d6*^+^) and fibroblasts (Fib, *Sparc*^+^). The top three marker genes for each cell type are shown in Fig. 1f (complete marker genes are shown in Supplementary Table S1).

We first focused on the changes in immune cells and found significant decreases in the B cell proportions in both tissues, while we found increased proportions of T cells in epithelial tissues (Fig. 1g, h). In immune cells, differential gene expression analysis revealed that genes associated with immune regulation, biotic stimulus, and inflammatory bowel disease (IBD) were significantly upregulated, including *Zfp36*, *Ptpn22*, and *Isg20*^17–19^ (Fig. 1i). Gene Ontology (GO) analysis also identified terms associated with immune activation, such as response to biotic stimulus (Fig. 1j).

Next, we noticed specific changes in the IECs of ALF mice. Unexpectedly, the proportion of stem cells was significantly decreased (*P* < 0.05), and the proportion of goblet cells was slightly increased (Fig. 1k). A total of 508 differentially expressed genes between the ALF group and control group (ALF_DEGs) were identified within IECs (log2FC > 0.25). Genes associated with stress, oxidative damage, and microbial infection response were significantly upregulated (e.g., *Saa1*, *Duoxa2*^20^ and *Slfn4*), which coincided with the results of the GO analysis of ALF_DEGs, suggesting that IECs were in an injured state (Fig. 1l, m). We also observed significant downregulation of *Add3*, a gene involved in interepithelial cell contacts^21^, which demonstrated disruption of intestinal epithelial tight junctions. In summary, these changes in immune cells and IECs create a picture of the intestine in ALF mice, showing dysregulation of the immune system and disruption of the intestinal epithelial barrier and microenvironment.

### Intestinal immune cells respond to ALF

**Fig. 2:**
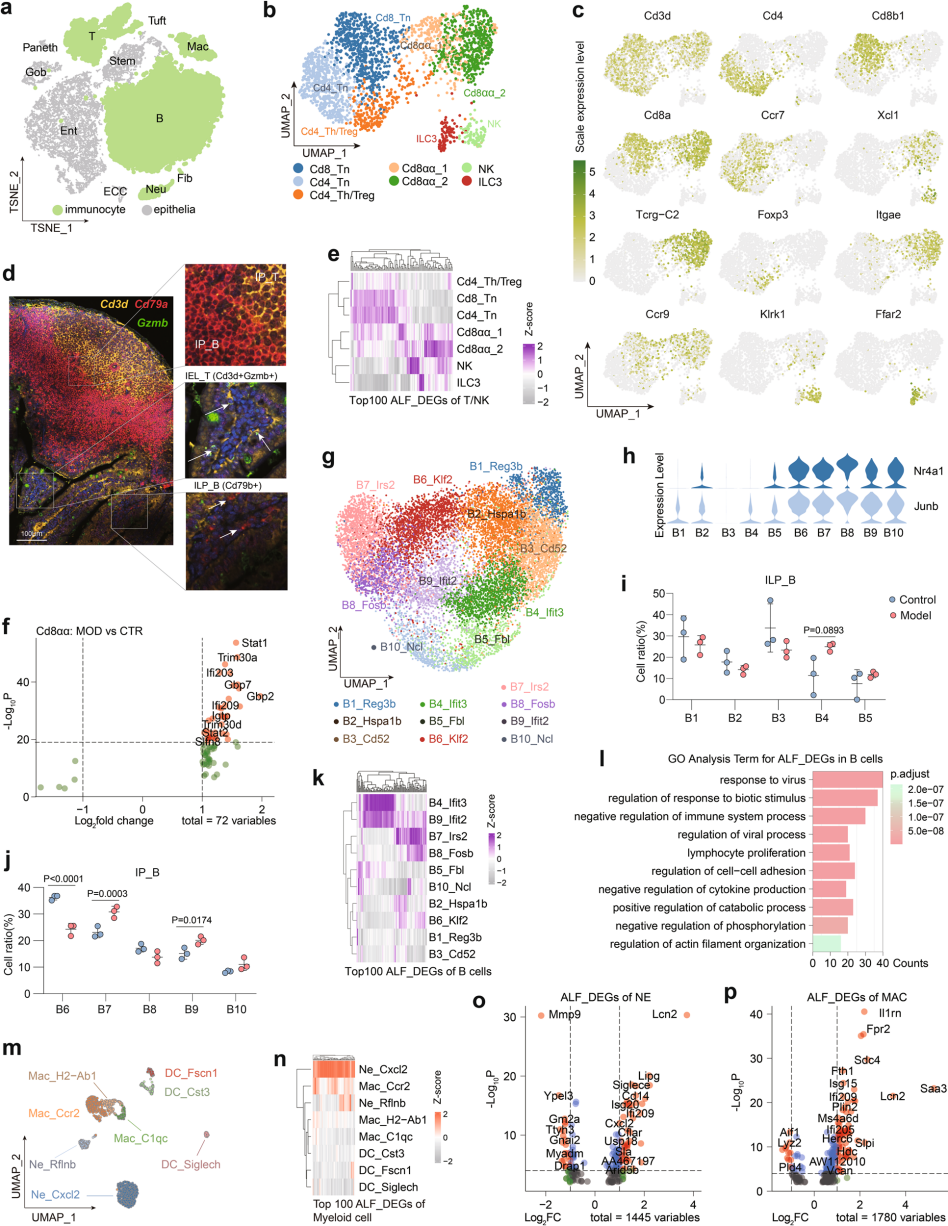
ALF induces intestinal immune cell activation. **a** UMAP plot of all immunocytes. **b** UMAP plot of seven T/NK cell clusters. **c** Featureplot of key T/NK cell markers used to describe T/NK cell cluster identity and link it to cell type. **d** Representative images of immunofluorescence (IF) staining for *Cd3d*, *Cd79a* and *Gzmb* in PPs and IE tissues. For all IF analyses, DAPI (blue) was used to stain the nuclei of the cells. Scale bar, 100 μm. **e** Clustered heatmap of the average expression of the top 100 ALF_DEGs in each T/NK cell cluster. **f** Volcano plot of ALF_DEGs in Cd8_αα cells. **g** UMAP plot of eight B cell clusters. **h** Violin plots showing the expression of *Nr4a1* and *Junb* in B cell clusters. **i** Percentages of ILP_B cell clusters. Means ± SD, two-way ANOVA. **j** Percentages of IP_B cell clusters. Means ± SD, two-way ANOVA. **k** Clustered heatmap of the average expression of the top 100 ALF_DEGs in B cells. **l** GO term enrichment analysis for upregulated ALF_DEGs in B cells. **m** UMAP plot of eight myeloid cell clusters. **n** Clustered heatmap of the average expression of the top 100 ALF_DEGs in myeloid cells. **o** Volcano plot of ALF_DEGs in neutrophils. **p** Volcano plot of ALF_DEGs in macrophages. Numbers of mice: model group (*n* = 3), control group (*n* = 3).

To further reveal the changes in the intestinal immune cells of ALF mice, we analyzed the heterogeneity of immune cells (including T/NK cells, B cells, and myeloid cells) (Fig. 2a).

#### T/NK cells

Unsupervised clustering was used to classify T/NK cells into seven clusters (Fig. 2b). Common T/NK cell subpopulations were identified based on the expression of recognized marker genes, such as Cd4_Tn and Cd8_Tn (*Ccr7* and *Sell*), Cd4_Treg/Th cluster (*Foxp3* and *Maf*) and NK (*Klrb1c* and *Klrk1*) subpopulations (Fig. 2b, c; Supplementary Fig. S1a). Alongside the identification of these common cell clusters, several specific T/NK cell clusters in the intestine were also identified. First, Cd8αα_1 and Cd8αα_2, both of which show a unique *Cd3d*^*+*^*Cd8a*^*+*^*Cd8b*^*–*^*Tcrg*^*–*^*C2*^*+*^ signature and high expression of the characteristic genes *Itgae* and *Ccr9* associated with tissue residence, were identified as intraepithelial T lymphocytes (IEL_T)^22^. Cd8αα_2 expressed increased *Gzma*, *Gzmb*, and *Ccl5* levels, with classical toxic T cell characteristics (Fig. 2b, c; Supplementary Fig. S1a). Finally, based on the specific expression of *Ffar2* and *Il22*, we identified an ILC3 cluster (type 3 innate lymphoid cells^23^) (Fig. 2b, c; Supplementary Fig. S1a).

We carefully examined the origin of the T/NK cells and found that the majority of T/NK cells captured within the IE were Cd8αα cells (including Cd8αα_1 and Cd8αα_2) (Supplementary Fig. S1b). The clustered heatmap drawn by mapping DEGs of control IE vs control PPs (tissue_DEGs) to cell clusters showed that the seven T/NK cell clusters could be divided into two main branches. Where Cd4_Th/Treg, Cd4_Tn and Cd8_Tn were the first branch, these clusters were more often derived from PPs and were identified as IP_T (intestinal PP T cells). The second branch, IEL, included clustered Cd8αα_1, Cd8αα_2, ILC3, and NK cells, which were mostly from the IE (Supplementary Fig. S1b, c). To verify the presence of IEL_T within the intestinal epithelial tissue, we performed IF assays. As shown in Fig. 2d, *Cd3d*^+^*Gzmb*^+^ T cells were present only at the base of the IE, but not observed within PPs (Fig. 2d).

We further explored the activation of T/NK cells by mapping the top 100 ALF_DEGs of T/NK cells to these cell clusters, and the clustered heatmap showed that IP_T and IEL had different activation genes in ALF mice (Fig. 2e). In addition, we found more ALF_DEGs in IEL than in IP_T (*n* = 1028 vs *n* = 571, log2FC > 0.25), indicating a greater impact of ALF on IEL, which may have been related to increased proportions of T cells in the IE (Fig. 1h; Supplementary Fig. S1d, e). In ALF mice, no changes were observed in the proportions of other cell clusters except for a slightly elevated proportion of the Cd8αα_2 cell cluster (Supplementary Fig. S1f, g). Given the specificity of such IELs, we further investigated the changes in Cd8αα cells, and these location-specific cell clusters upregulated many genes associated with interferon responses (*Ifi209* and *Stat1*, etc.), which implies the presence of an underlying microbial infection in the intestine (Fig. 2f).

#### B cells

Based on the unsupervised clustering, cluster DEGs and subsequent GO analysis, we defined ten B cell clusters in the mouse intestine (*n* = 13,778) (Fig. 2g). B1 was characterized by high expression of *Reg3b* (an antimicrobial peptide), *Lypd8* (involved in inhibition of microbiota adhesion to IECs^24^), and *Zg16* (a mucus component), and B1 was enriched for GO terms associated with cell killing and microbial resistance (Fig. 2g; Supplementary Fig. S1h, i). Thus, B1 may be the B cell cluster associated with intestinal microbial resistance. Then, IE-associated clusters of unactivated B cells were identified due to B2-specific expression of *Add3* (involved in epithelial cell attachment^21^) and *Hspa1b* (a marker for unactivated B cells). High expression of *Cd52* (an essential gene for intestinal homeostasis^25^) and *Vpreb3* (a pre-B cell marker gene) was used to identify B3 as the pre-B cell population in the intestine. B4 and B9 were two interferon-activated B-cell clusters that highly expressed genes associated with interferon activation (*Ifit3*, *Slfn5*, and *Gbp7*) and were enriched for viral response/cellular stress-related GO terms. B5 and B10 had high expression of *Fbl*, *Ncl*, *Shmt2*, and *Ccnd2* and genes related to cycle and RNA synthesis; B5 and B10 were identified as two clusters of B cells with high proliferative activity. B6 was characterized by high expression of the follicular B cell marker genes *Klf2*^26^ and *Hspa1b* and was therefore identified as an unactivated follicular B cell cluster. B7 and B8, which highly expressed *Irs2* (a B cell-activation marker^27^), *Ccr7* (involved lymphatic migration^28^), and *Nr4a1* (tissue-resident), were identified as two activated tissue-resident B cell clusters (Fig. 2g; Supplementary Fig. S1h, i).

To classify the numerous B cell clusters, two different clusters of interferon activation characteristics (B4 and B9) were compared. The results showed that B6-B10 highly expressed *Nr4a1* and *Junb* (Fig. 2h). Consistent with these findings, after tissue_DEGs of B cells were mapped to cell clusters, the clustered heatmaps showed that intestinal B cells could be divided into two main branches, namely, IP_B (containing intestinal PP B cells with elevated *Nr4a1* and *Junb* expression) and ILP_B (containing intestinal lamina propria B cells in epithelial samples with low expression of *Nr4a1* and *Junb*) (Fig. 2h; Supplementary Fig. S1j). The presence of B cells in PPs and lamina propria B cells was also supported by the results of IF staining of *Cd79b* in PPs and the adjacent IE, as shown in Fig. 2d.

In ILP_B, the proportions of B2 and B3 were significantly decreased, while the proportion of B4 was significantly increased (*P* < 0.05). In IP_B, the proportion of B6 was significantly decreased, whereas the proportions of B7 and B9 were significantly increased (*P* < 0.05). These changes in the proportion of B cell clusters confirmed that ALF-induced intestinal B cell activation (Fig. 2i, j). To explore the activation levels of B cells in different locations in the intestines of mice with ALF, we further mapped the top 100 ALF_DEGs of B cells to these cell clusters, and the clustered heatmap showed that B4, B9, B7, and B8 were the four B cell clusters most affected by ALF (Fig. 2k). These B cell clusters were characterized by high expression of *Ifit3* and *Irs2* and showed a high correlation with the inflammatory response; this result represented the similar responsiveness of B cells to ALF in different ecological locations. Finally, among all B cells, we identified 266 B cell ALF_DEGs (log2FC > 0.25) and GO terms associated with immune activation (Fig. 2l; Supplementary Fig. S1k). In summary, these analyses indicate the activation of intestinal B cells after ALF induction.

#### Myeloid cells

The macrophages and neutrophils in the initial clusters were further divided into eight clusters, including two neutrophil clusters, three macrophage clusters, and three dendritic cell (DC) clusters (Fig. 2m). First, among neutrophils, Ne_Rflnb cells with high expression of *Syne1*, *Ccr3*, *Rflnb* and *Retnlg* (classic neutrophil markers^29^) were identified as unactivated neutrophils. Due to the specific high expression of *Cxcl2*, Ne_Cxcl2 was identified as an activated neutrophil cluster (Supplementary Fig. S1m)^5^. Among the three macrophage clusters, Mac_Ccr2 with high expression of *Ccr2* (involved in cell migration and inflammation) was identified as a cluster of activated macrophages. Mac_H2-Ab1 highly expressed *H2-Ab1* and *C1qc*, the classic unactivated macrophage markers. Mac_C1qc highly expressed *C1qc* and *Apoe*. *Apoe*^+^ macrophages have previously been reported to be associated with chronic inflammation^30^; thus, Mac_C1qc was defined as an inflammation-associated macrophage cluster. Among the DC clusters, DC_Cst3 was classified as cDC1 due to high expression of the cDC1 cell marker genes *Cst3* and *Wdfy4*^30,31^, while DC_Fscn1 specifically expressed *Cst3* and *Fscn1* and was classified as a subpopulation of cDC1 with high migration capacity^31^. DC_Siglech highly expressed *Siglech* and *Ly6d* and was therefore classified as pDC^22^ (Supplementary Fig. S1m).

Among the various myeloid cell clusters, the proportion of Ne_Cxcl2 was significantly increased in ALF mice (Supplementary Fig. S1l). The top 100 ALF_DEGs of myeloid cells were mapped to these cell clusters, and the clustered heatmap revealed more significant changes in neutrophils and macrophages relative to DCs in ALF mice, especially in the Ne_Cxcl2 cluster (Fig. 2n). Both neutrophils and macrophages showed upregulation of some genes related to pathogen resistance/interferon response/infection in ALF mice (*Lcn2*, *Ifi209*, and *Saa3*), indicating that ALF leads to activation of myeloid cells in the intestine (Fig. 2o, p).

In summary, our data demonstrated that in ALF mice, intestinal immune cells, including T/NK cells, B cells, and myeloid cells, are rapidly activated. For T cells, we demonstrated that the intestinal IELs are highly responsive to ALF. The increased number of T cells in the IE suggests a microbially infected state of the intestine; as previously reported, adenovirus infection promotes IEL_T recruitment^32^. For B cells, we identified a cluster of B cells characterized by the secretion of antimicrobial peptides, revealed differential expression of *Nr4a1* and *Junb* by B cells within the PPs and the lamina propria of the intestine, and found that B cells in different ecological locations shared similar activation patterns. The decrease in B cells in the intestine may have been caused by microbial infection^33^ or by the migration of B cells from the PPs to the liver in response to liver injury. In myeloid cells, activation of macrophages and neutrophils was also characterized. The intestine is considered to be the largest immune and endocrine organ of the digestive system, and it plays a central role in regulating systemic immune homeostasis^34,35^. These dysregulated immune cells may further induce intestinal mucosal immune disorders and exacerbate intestinal barrier damage, subsequently releasing excessive cytokines and other signals into circulation and accelerating the process of ALF in mice.

### IECs response to ALF

As a crucial component of the intestinal epithelial barrier that strongly interacts with the immune system and the intestinal microbiome, IECs are also important players in ALF. To further reveal the changes in IECs in ALF mice, we analyzed the heterogeneity of enterocytes, intestinal stem cells, and specific IECs (including goblet cells, endocrine cells, Paneth cells, and tuft cells) in the intestine.

#### Enterocytes

**Fig. 3:**
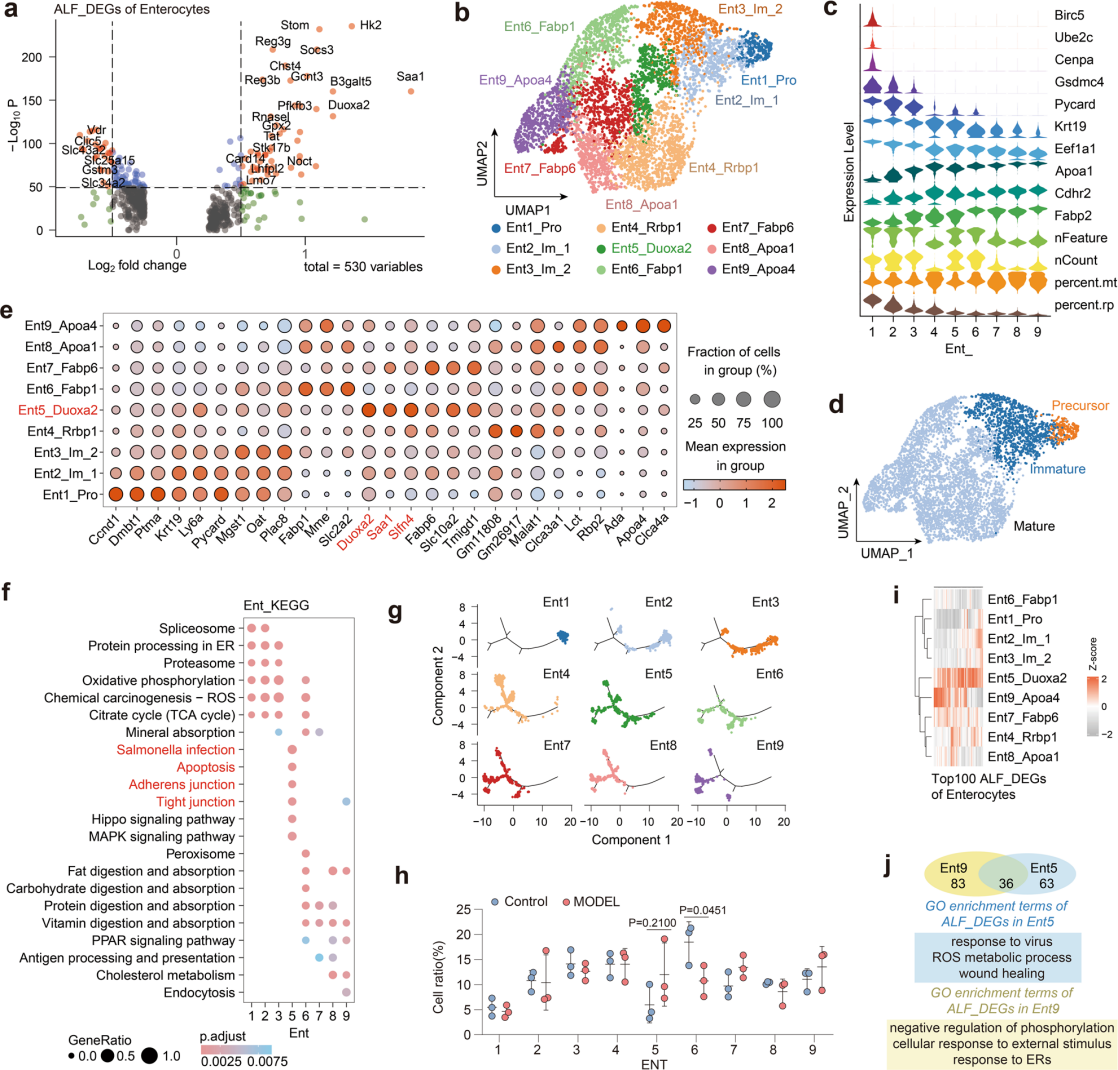
Injured enterocyte cluster in the intestines of D/L-induced ALF mice. **a** Volcano plot of ALF_DEGs in enterocytes. **b** UMAP plot of nine enterocyte clusters. **c** Violin plot drawn for marker genes and basic quality control information for three levels of maturation of enterocytes. **d** UMAP plot of three levels of maturation of enterocytes. **e** Dot plot of gene expression for the top three marker genes of each enterocyte cluster. **f** KEGG analysis of enterocyte cluster marker genes. **g** Pseudotime axis of enterocytes with enterocyte clusters distributed on the axes. **h** Percentages of enterocyte clusters. Means ± SD, two-way ANOVA. **i** Clustered heatmap of the average expression of the top 100 ALF_DEGs in enterocytes. **j** Venn diagram of ALF_DEGs and GO terms enrichment analysis of Ent5 and Ent9. Numbers of mice: model group (*n* = 3), control group (*n* = 3).

First, we identified 530 ALF_DEGs in enterocytes (log2FC > 0.25) (Fig. 3a). Compared to the control group, there were dramatic changes in the enterocytes of ALF mice, and genes associated with intestinal microbes, absorption, and inflammation, e.g., *Saa1, Hk2* (promoting epithelial microbial infection^36^), *Stom* (regulating ion transport), *Socs3* (promoting IBD progression^37^) and *Reg3g* (intestinal antimicrobial peptide), were significantly upregulated in the enterocytes of ALF mice (Fig. 3a). To further characterize the heterogeneity of enterocytes in ALF mice, we analyzed enterocytes in depth (*n* = 5300). Unsupervised clustering was used to divide the enterocytes into nine clusters (Ent1 to Ent9) (Fig. 3b). Based on marker genes for maturation status^38^, enterocytes were subdivided into the following groups: precursor enterocytes (with high expression of *Birc5*, *Ube2c*, and *Cenpa*), immature enterocytes (with high expression of *Gsdmc4*, *Picard*, *Krt19*, and *Eef1a1*) and mature enterocytes (with high expression of *Apoa1*, *Cdhr2*, and *Fabp2*) (Fig. 3c, d). Among these, mature enterocytes exhibited abundant heterogeneity. Ent4_Rpbp1 only highly expressed genes related to protein synthesis/RNA translation process-related pathways and was not enriched in the intestinal absorption-related KEGG pathway; therefore, Ent4_Rpbp1 may be a specific cluster of mature enterocytes not involved in intestinal absorption. Ent6_Fabp1 highly expressed *Slc2a2* (a glucose transporter^39^) and *Fabp1*, and the associated DEGs were uniquely enriched in the peroxisome-associated KEGG pathway and have thus been identified as a cluster of mature enterocytes involved in absorption and antioxidative effects. The Ent7_Fabp6 cluster highly expressed *Fabp6*, *Tmigd1* (cell adhesion molecules for intestinal brush border formation^40^), and *Slc10a2* (a marker of intestinal bile acid uptake by apical cells of the distal ileum^41^), and the associated DEGs were enriched in the KEGG pathways associated with inorganic salt, protein, and vitamin absorption and was therefore identified as a classical cluster of absorptive mature enterocytes in the distal ileum. Ent8_Apoa1, which specifically expressed *Rbp2* (which promotes vitamin A absorption^42^), *Lct* (which regulates intestine microbiological composition^43^), and *Clca3a1* (which controls intestinal mucus^44^), was identified as a cluster of mature enterocytes associated with intestinal mucosal homeostasis (Fig. 3c–f).

Notably, two enterocyte clusters exhibited a higher response to ALF. Based on the high expression of *Duoxa2* (a marker of inflammatory intestinal oxidative damage^20^), *Saa1* (inflammatory stress-related), and *Slfn4* (inflammation-related), and the specific enrichment of microbial infection, tight junction, and apoptosis-related KEGG pathways, Ent5_Duoxa2 was defined as an injured enterocyte cluster (Fig. 3c, d). Ent9_Apoa4, which highly expressed *Clca4a* (enterocyte apical membrane protein^45^) and *Ada* (an IBD candidate biomarker^46^) and was enriched in pathways associated with microbial infection and endocytosis, was identified as a cluster of mature enterocytes involved in mucosal immunity (Fig. 3c–f).

The process of renewal and differentiation of the IE has been extensively studied^47^. We hypothesized that ALF, which progresses rapidly over several hours, cannot cause differentiation changes in enterocytes. To put it simply, the clusters of injured enterocytes should be independent of enterocyte differentiation. Therefore, Monocle 2.0 was used to analyze the differentiation trajectory of enterocytes to verify this hypothesis^48^. Consistent with the expectations, as immature enterocytes, Ent1/2/3 occupied the initiating branches of the pseudotime axis, while Ent4/6/7/8/9 were located in the posterior branches of the pseudotime axis; this result was consistent with the consensus that precursor cells gradually differentiate into mature enterocytes (Fig. 3g; Supplementary Fig. S2a, b). In addition, we found that during enterocyte maturation, the activated pathway shifts first from protein translation to energy metabolism, then to intestinal absorption, and finally to tight junctions and intestinal barrier function, suggesting that the shift in energy metabolism may promote maturation of enterocytes (Supplementary Fig. S2c). Crucially, Ent5 is poorly associated with the pseudotime axis and scattered in different states, which indicates that Ent5 is a cell cluster that is induced by specific intestinal environmental stimuli rather than resulting from enterocyte differentiation (Fig. 3g; Supplementary Fig. S2a, b).

Furthermore, the proportion of Ent5 (injured enterocytes) was elevated, accompanied by a significant decrease in the proportion of Ent6 (typical resorbing enterocytes) after ALF (Fig. 3h). We identified ALF_DEGs of enterocytes and found that most of the ALF_DEGs were highly expressed by Ent5 and Ent9 clusters specifically (Fig. 3i). Further differential analysis revealed that Ent5 and Ent9 shared ~1/3 of the ALF_DEGs, with Ent5 being more notably linked to changes associated with microbial infection, oxidative stress, tissue damage, apoptosis, and cell adhesion than Ent9 (Fig. 3j). Moreover, Ent9 showed a better fit to the proposed pseudotime differentiation, which implies that Ent9 may be associated with the end state of intestinal differentiation (the mucosa-associated intestinal barrier) (Fig. 3g; Supplementary Fig. S2a, b). SCENIC analysis revealed Ent5-specific activation of inflammation-related transcription factors (TFs), such as *Irf2*, *Irf6*, and *Cebpd*, and Ent9-specific activation of *Pbx3* (a celiac disease-related gene^49^) (Supplementary Fig. S2d). These results support the hypothesis that Ent5 is a cluster of injured enterocytes that is closely associated with ALF-induced intestinal epithelial damage, while Ent9 may be a cluster of enterocytes associated with the intestinal barrier response.

Alterations in the intestinal microenvironment in ALF mice may induce changes in cellular communication patterns^50^. To reveal the differences in communication between the injured enterocytes and other cell types, we scrutinized the changes in cellular communication during ALF. We found that in ALF mice, information flow in eight signaling pathways was decreased (via SOMATOSTATIN, GAS, KIT, etc.), while that in twelve signaling pathways was increased (via GALECTIN, CXCL, CCL, etc.) (Supplementary Fig. S3a). By dividing the enterocyte clusters into injured enterocytes (Ent_inj, including Ent_5) and normal enterocytes (Ent_nor, including Ent_1/2/3/4/6/7/8/9), we further analyzed the associations between the pathways upregulated in ALF and injured enterocytes. We found that Ent_inj communication was enhanced by ALF in five signaling pathways (involving EGF, VISFATIN, TNF, BMP, and PARs) (Supplementary Fig. S3b–f). Among the five signaling pathways, the VISFATIN pathway has been reported to promote intestinal inflammation, the PARs pathway is associated with inflammatory injury in IBD, and the TNF pathway is crucial in cell death. Activation of these pathways was inextricably linked to the activation of immune cells in Ent_inj cells of ALF mice (Supplementary Fig. S3b–d). In contrast, activation of the EGF pathway and BMP pathway (which promotes epithelial differentiation under inflammatory conditions^51^), which constitute the communication between IECs, may be a repair signal from Ent_inj (Supplementary Fig. S3e, f). In short, Ent_inj cells exhibit significantly different communication pathways compared to Ent_nor cells, and these differential cellular communication pathways can be further activated by ALF. Taken together, these findings identify a cluster of injured enterocytes that exhibited significantly different communication pathways compared to Ent_nor clusters in ALF mice and suggest that specific targeting and repair of these cells may relieve IE damage and thus improve intestinal ecology in ALF.

#### Intestinal stem cells

**Fig. 4:**
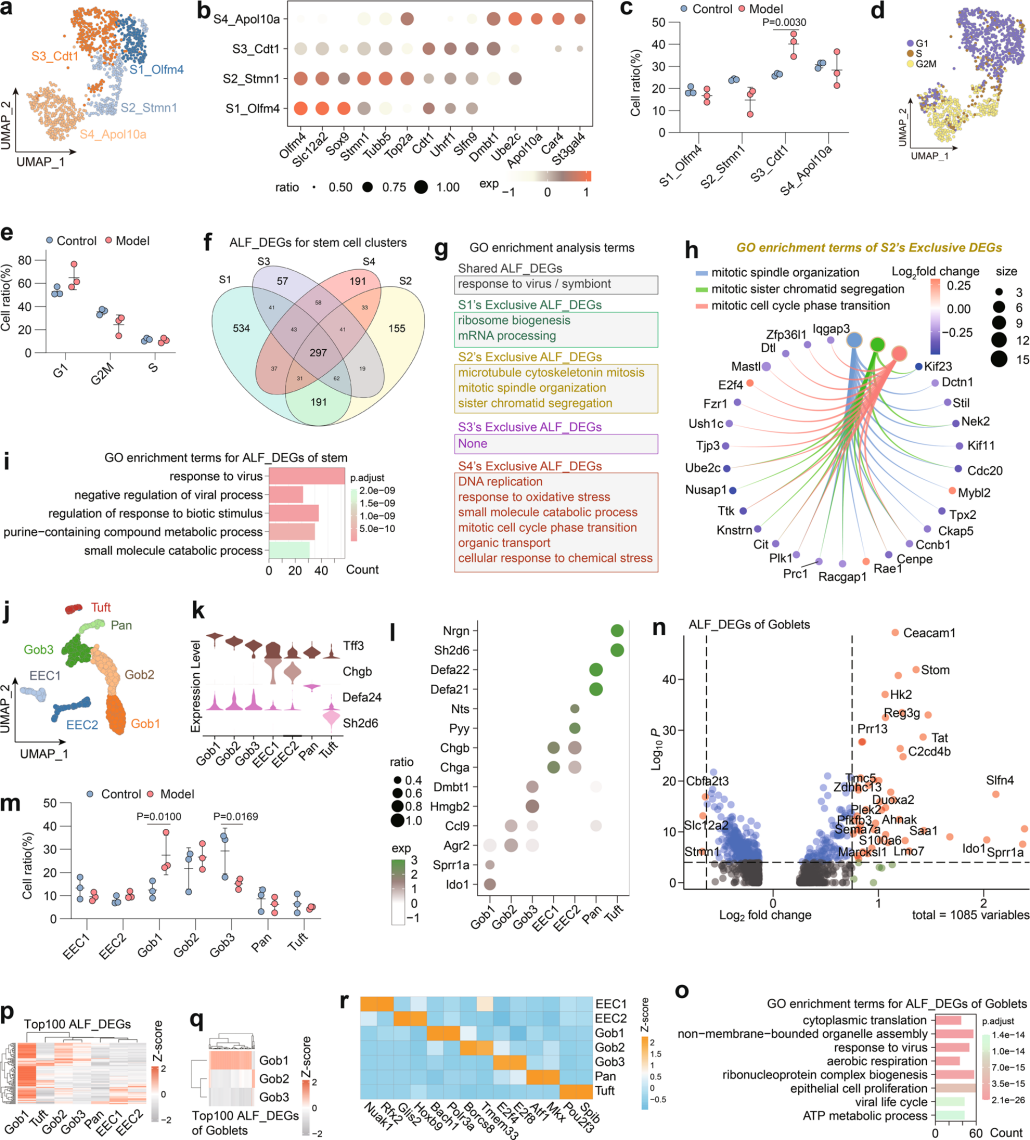
ALF induces intestinal stem cell cycle arrest and goblet cell activation. **a** UMAP plot of four intestinal stem cell clusters. **b** Dot plot of the top four marker genes of the intestinal stem cell clusters. **c** Percentages of intestinal stem cell clusters. Means ± SD, two-way ANOVA. **d** UMAP plot colored by cell cycle phase based on the results of Scran analysis. **e** Percentages of intestinal stem cell cycles. Means ± SD, two-way ANOVA. **f** Venn diagram of ALF_DEGs drawn for each intestinal stem cell cluster. **g** GO term enrichment analysis for ALF_DEGs of each intestinal stem cell cluster. **h** Expression of genes related to mitosis in S2 between the control and model mice. **i** Top five terms in the GO term enrichment analysis for the ALF_DEGs in intestinal stem cells. **j** UMAP plot of specific IECs. **k** Violin plots showing key cell markers used to describe goblet cells, EECs, Paneth cells and tuft cells and link them to cell type. **l** Dot plot of the top two marker genes of specific IECs clusters. **m** Percentages of specific IECs clusters. Means ± SD, two-way ANOVA. **n**, **o** Volcano plot and top five terms in the GO term enrichment analysis for ALF_DEGs in goblet cells. **p**, **q** Clustered heatmap of the average expression of the top 100 ALF_DEGs in specific IECs/goblet cells. **r** Heatmap drawn for TFs specifically activated in specific IECs clusters. Number of mice: model group (*n* = 3), control group (*n* = 3).

Renewal of IECs is performed by intestinal stem cells located at the base of the crypts, and repair of the damaged intestine is also highly dependent on these cells, which maintain continuous proliferative activity^52^. We hypothesized that intestinal stem cells may be disturbed by microenvironmental changes and lose their proliferative capacity of repairing the injured intestine. Based on unsupervised clustering, stem cells (*n* = 1352) were subdivided into four clusters (S1 to S4) (Fig. 4a). In detail, cells in the S1_Olfm4 cluster expressed the most classical intestinal stem cell marker *Olfm4*^38^, and therefore this cluster was identified as the most typical intestinal stem cells. S2_Stmn1, which highly expressed *Olfm4* while specifically expressing some cycle-related genes, such as Stathmin1 (*Stmn1*) and β-microtubulin (*Tubb5*), was identified as a mitotic stem cell cluster. High expression of *Cdt1* (a component of the G1 phase prereplication complex^53^) and *Uhrf1* (highly expressed in late G1 phase^54^) was used to classify S3_Cdt1 as a late G1 stem cell cluster. S4_Apol10a highly expressed *Top2a*, *Apol10a*, *Car4* (carbonic anhydrase IV), and *Ube2c* (a precursor intestinal cell marker), and these cells belonged to a stem cell cluster that has acquired partial IECs function (Fig. 4b).

During ALF, the proportion of the S1 cluster was largely stable, while the proportions of S2 and S4 decreased; these decreases were accompanied by a significant increase in the proportion of S3 (Fig. 4c). Based on the cell cycle analysis with Scran, the model group exhibited more G1 phase cells and fewer G2/M phase cells than the control group (Fig. 4d, e). To further explore the heterogeneity of intestinal stem cells clusters in ALF, we analyzed the ALF_DEGs of individual stem cell clusters (Fig. 4f). GO analysis of these DEGs revealed that each cell cluster was affected by immune activation, and S2 and S4 were uniquely enriched in a large number of cell cycle/mitotic-related terms, especially S2 (Fig. 4g). Further analysis revealed that many classical cell cycle/mitosis-related genes in S2 were downregulated in ALF mice, such as *Kif23* and *Plk1*, suggesting that ALF induces mitotic inhibition in intestinal stem cells (Fig. 4h). GO analysis of ALF_DEGs in intestinal stem cells indicated enrichment in terms associated with immune response/interferon/viral response, suggesting abnormal immune activation in the intestine, which induced intestinal stem cells dysfunction (Fig. 4i). In conclusion, our analysis shows that ALF induces intestinal stem cells cycle arrest, which may abolish epithelial repair.

#### Specific IECs

We analyzed the expression of classic marker genes of specific IECs and reclassified specific IECs into seven clusters, including three goblet cell clusters (*Tff3*^+^), two EEC clusters (*Chgb*^+^), one Paneth cell cluster (*Defa24*^+^), and one tuft cluster (*Sh2d6*^+^) (Fig. 4j, k). The goblet cell cluster included Gob1 with high expression of *Ido1* (associated with intestinal barrier mucus^55^) and *Sprr1a* (associated with tissue damage repair^56^). Gob2 had high expression of *Agr2* (a gene essential for mucus barrier function^57^) and the chemokine *Ccl9*. Gob3 had high expression of cell cycle-related genes such as *Hmgb2* and *Dmbt1*. The two EEC clusters included EEC1 with high expression of *Chgb* and *Chga* and EEC2 with high expression of the classic marker genes *Pyy*^58^ and *Nts* (Fig. 4l). EEC1 and EEC2 were similar to the previously reported ECM (intestinal chromosome) and L cell clusters, respectively^59^.

After analysis of all the special IECs, we found that the proportion of Gob1 was significantly increased while that of Gob3 was significantly decreased in ALF mice (*P* < 0.05) (Fig. 4m). ALF led to the activation of goblet cells, and genes such as *Hk2*^36^, *Slfn4*, *Stom*, and *Duoxa2*^20^ were highly expressed in ALF mice. The results of GO analysis also highlighted terms associated with viral/immune responses/oxidative stress (Fig. 4n, o). Most of the ALF_DEGs in specific IEC types were specifically highly expressed in Gob1 (Fig. 4p, q), which indicated a highly activated state of Gob1 cells. Further TF analysis revealed high activity of *Bach1*, a TF associated with oxidative stress injury, in Gob1 cells, which also supports the relevance of Gob1 to intestinal injury^22^ (Fig. 4r). As goblet cells are essential for intestinal homeostasis and the intestinal barrier^60^, our results suggest that the disruption of the intestinal barrier in ALF is accompanied by goblet cell activation and that specific inhibition of the goblet cell-activation cluster may help protect the IE.

### *Ep300* is a shared upstream activator, and its inhibition attenuates ALF

**Fig. 5:**
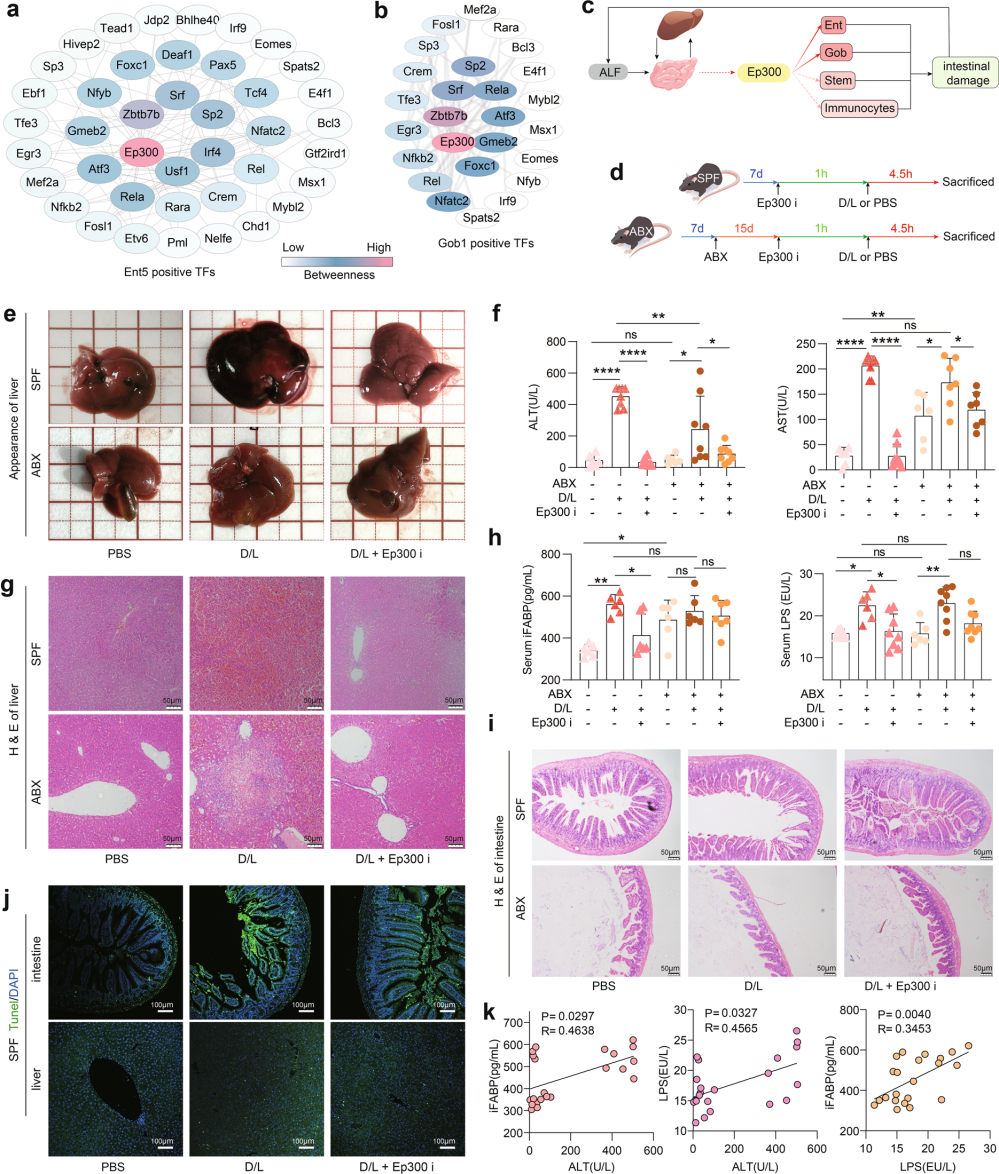
*Ep300* inhibition attenuates intestinal and hepatic injury in D/L-induced ALF mice. **a** Analysis of the protein–protein interaction network for specific activation of TFs in Ent5 via STRING-Cytoscape. **b** Analysis of protein–protein interaction networks in Gob1 that specifically activate TFs via STRING-Cytoscape. **c** Schematic diagram of the mechanism of *Ep300* regulation of intestinal cells. **d** Animal experimental design. **e** Representative images of the mice livers in each experimental group. **f** Activity of AST and ALT in mouse serum in the presence or absence of ABX, D/L and *Ep300*i (means ± SD, two-way ANOVA, ns, no significant difference. *P < 0.05. **P < 0.01. ***P < 0.001. ****P < 0.0001, *n* = 6–8 mice per group, same as below). **g** Representative pictures of H&E staining of the mouse liver. Scale bars, 50 μm. **h** Levels of iFABP and LPS in mouse serum in the presence or absence of ABX, D/L and *Ep300*i. **i** Representative pictures of H&E staining of the mouse intestine. Scale bars, 50 μm. **j** Representative pictures of TUNEL staining of the mouse liver and intestine in the presence or absence of D/L and *Ep300*i. Scale bars, 100 μm. **k** Correlation analysis of serum ALT, iFABP and LPS in mice.

In general, intestinal barrier disruption caused by alterations in IECs precedes intestinal immune system activation during the progression of ALF^61^. In all the altered cell profiles, we found that the most dramatically altered IECs, including enterocytes, intestinal stem cells, and goblet cells, have similar activation patterns, suggesting that they may share the same transcriptional activation program, which may be an early driving factor in the development of ALF. Thus, we screened TFs activated in Ent5 but relatively inactive in the immature enterocytes in Ent1/2/3 (for protecting intestinal differentiation) and used STRING-Cytoscape to map the activated TFs to protein–protein interaction networks and for interaction strength analysis. The results showed that *Ep300* is the key TF for the pathological activation of enterocytes (Fig. 5a). Then, we screened the TFs activated in Gob1 cells (inactive in Gob2 and Gob3) and analyzed the protein–protein interaction network and interaction strength along the same lines. We found that *Ep300* was also a key TF for activation in Gob1 cells (Fig. 5b). In addition, it has been reported that *Ep300* inhibition promotes intestinal stem cell-mediated crypt regeneration by regulating the progression of the IECs cycle^62^. Further, we analyzed the changes in *Ep300* expression in individual cell clusters. As shown in Supplementary Fig. S4a–f, the expression level of *Ep300* was higher in the model group than in the control group in the vast majority of cell clusters across all cell types. Therefore, we hypothesized that inhibition of *Ep300* may ameliorate intestinal epithelial damage by inhibiting IECs injury, thus slowing or stopping the progression of ALF in mice (Fig. 5c). Furthermore, based on the inflammatory response and microbial infection status of the intestine in ALF mice, we hypothesized that the activation of *Ep300* may be associated with changes in intestinal microbes.

We thus tested the protective effect of an *Ep300* inhibitor (*Ep300*i), SGC-CEP30 (50 mg/kg^62^), against D/L-induced ALF in normal (specific pathogen-free, SPF) mice and antibiotic-treated (ABX) mice (Fig. 5d). As shown in Fig. 5e, *Ep300*i significantly inhibited D/L-induced liver injury and abnormal liver congestion. Furthermore, *Ep300*i significantly reduced serum alanine transaminase (ALT) and aspartate aminotransferase (AST) levels in SPF mice. In contrast to those in SPF mice, the degree of liver injury and ALT levels were reduced in ABX mice, and the protective ability of *Ep300*i was relatively diminished (Fig. 5e–g). Additionally, we found that in SPF mice, *Ep300*i attenuated D/L-induced intestinal damage and protected the intestinal barrier of mice, thereby reducing serum levels of intestinal fatty-acid binding protein (iFABP, an indicator of intestinal damage) and LPS (a microbial product). In contrast to the case in SPF mice, in ABX mice, *Ep300*i failed to suppress D/L-induced intestinal injury and elevated serum iFABP and LPS levels (Fig. 5h, i). Cell death in the intestine and liver was determined by TUNEL staining, and *Ep300*i treatment significantly reduced the number of dead cells, in both the liver and intestine of SPF mice (Fig. 5j). More critically, we found significant correlations among the serum levels of ALT, iFABP and LPS in mice, implying that intestinal injury and liver injury are inextricably linked in ALF mice (Fig. 5k).

Together, these results confirm that *Ep300* is an effective target to alleviate ALF. Furthermore, the lower ALF responsiveness and weaker *Ep300*i efficacy in ABX mice compared to SPF mice suggest that intestinal microbiome depletion is beneficial in reducing intestinal and liver damage from ALF. These results also show that, after intestinal microbiome depletion, the intestinal protective effect of *Ep300*i is completely lost and that the hepatoprotective effect is only somewhat attenuated after intestinal microbiome depletion. This means that the anti-ALF effect of *Ep300*i may be intestinal microflora independent, but the intestinal protective effect must be related to the intestinal microflora. This result is consistent with the intestinal origin of the cell population analyzed in our study and the role of the intestinal microbiota in ALF and suggested that we should focus on the intestine to explore the mechanism of *Ep300*i.

### *Ep300*i attenuates ALF by targeting the dysregulated clusters of IECs via the P38-JNK pathway

**Fig. 6:**
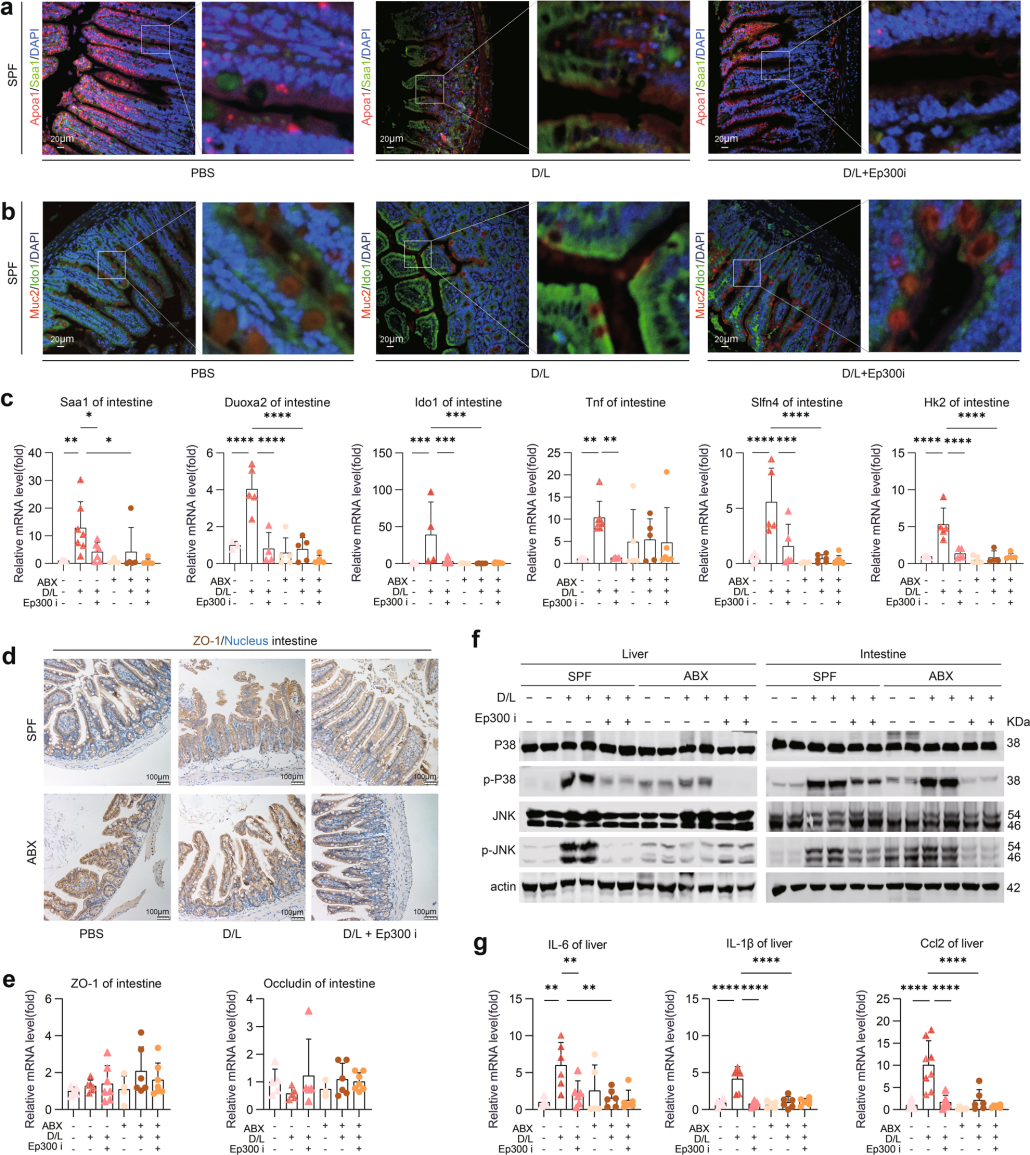
*Ep300* inhibition attenuates ALF in mice by inhibiting specific cell cluster changes via the P38-JNK pathway. **a**, **b** Representative IF images of *Apoa1*, *Saa1*, *Muc2* and *Ido1* in the mouse ileum. Scale bars, 20 μm. **c** q-PCR analysis of mRNA levels in mouse intestinal (means ± SD, two-way ANOVA. *P < 0.05. **P < 0.01. ***P < 0.001. ****P < 0.0001, *n* = 5–7 mice, same as below). **d** Representative immunohistochemistry (IHC) images of *ZO-1* in the mouse intestine. Scale bars, 100 μm. **e** q-PCR analysis of *ZO-1* and *Occludin* mRNA levels in mouse intestinal. **f** P38, p-P38, JNK and p-JNK expression in mouse liver and intestinal, as analyzed by western blotting assay. **g** q-PCR analysis of mRNA levels in mouse liver.

We next explored the relationship between the efficacy of *Ep300*i against ALF and intestinal epithelial alterations. First, we observed the in situ expression of key cell clusters by IF assay. As shown in Fig. 6a, *Saa1* expression was elevated in ALF mice compared to control mice and colocalized with enterocytes (*Apoa1*^*+*^), while *Ep300*i inhibited this trend. Similarly, the intestines of healthy mice exhibited lower *Ido1* expression in the intestine, and goblet cells (*Muc2*^*+*^) were arranged between enterocytes; in contrast, the intestines of ALF mice exhibited upregulated *Ido1* expression and enhanced colocalization of *Ido1* with *Muc2*. In addition, *Ep300*i inhibited this pathological change (Fig. 6b). Importantly, *Ep300*i reduced the mRNA expression levels of key genes (*Saa1*, *Duoxa2*, *Ido1* and *Hk2*) in intestinal tissues (Fig. 6c). Furthermore, we found that the gut-protective effect of *Ep300*i was independent of the regulation of intestinal tight junction proteins. Immunohistochemical staining of *ZO-1* showed no significant change in tight junctions after ALF; this finding was also confirmed by the absence of significant differences in intestinal *ZO-1* and *Occludin* mRNA levels (Fig. 6d, e). These results indicate that in the mouse IE, *Ep300*i exerts an anti-ALF effect by directly inhibiting the activation of IECs rather than promoting the expression of intestinal tight junction proteins. Western blotting analysis showed that *Ep300*i significantly inhibited P38 and JNK phosphorylation in the intestine and liver, both of which control closely related cascades of inflammatory responses, cellular stress and apoptosis; this effect was influenced by gut microbial depletion in ABX mice (Fig. 6f). As downstream effects, *Ep300*i reduced the mRNA levels of *TNF* and *Slfn4* in intestinal tissues and those of *IL-6*, *Il-1β* and *Ccl2* in the livers of ALF mice, respectively (Fig. 6c, g). These data confirm that *Ep300*i does not act through the classical enteroprotective mechanism of upregulating tight junction proteins but rather protects the gut against ALF by targeting the dysregulated clusters of IECs through the P38-JNK pathway.

### *Ep300* inhibition reshapes the intestinal microbial environment in ALF mice

**Fig. 7:**
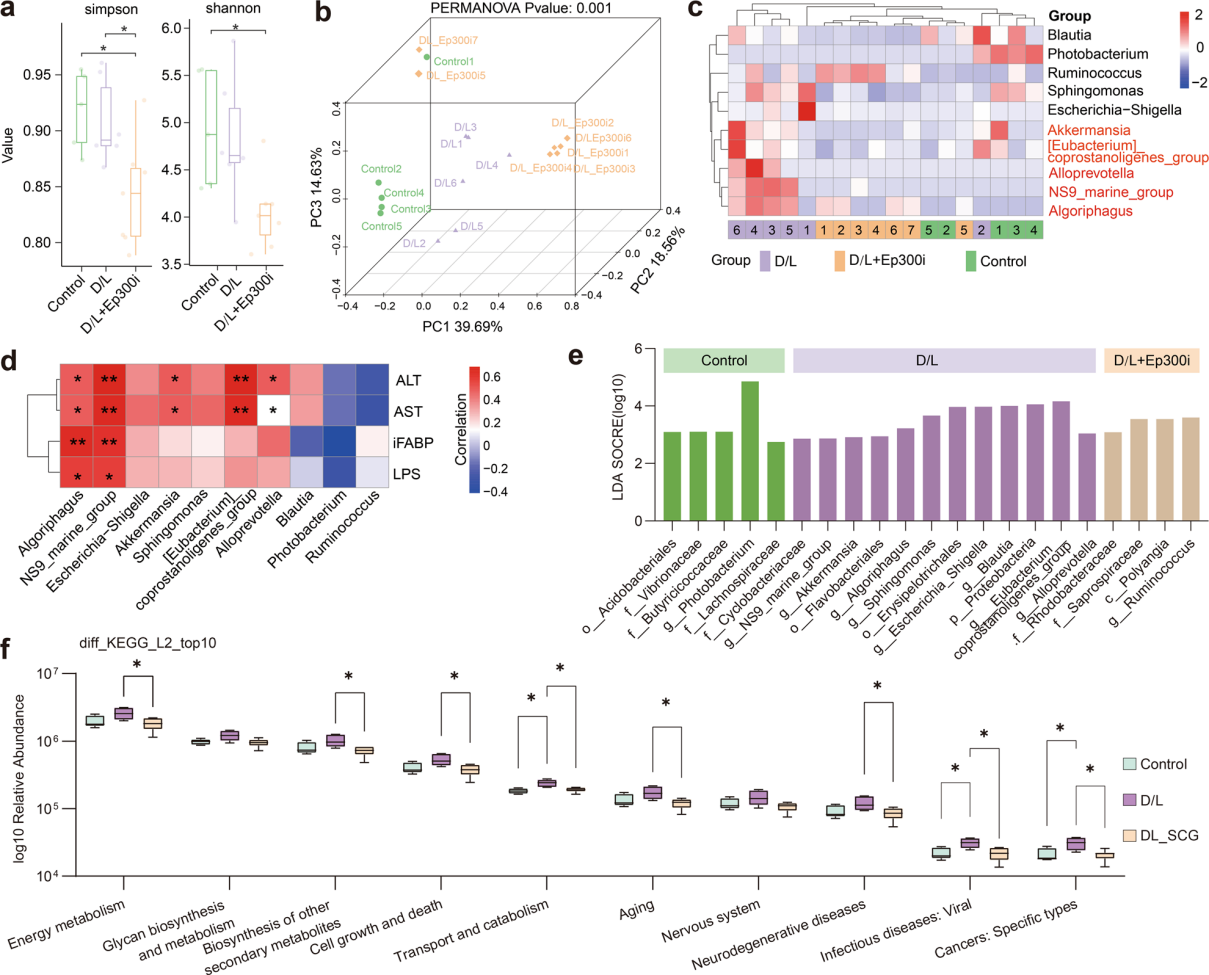
*Ep300* inhibition regulates intestinal microorganism homeostasis. **a** Box plot showing the differences in Simpson and Shannon indices between groups (Wilcoxon rank sum test. *P < 0.05). **b** 3D PCoA plot based on the relative abundance of amplicon sequence variants (ASVs) showing bacterial structural clustering. **c** Heatmap of the relative abundance of species differing at the genus level (Kruskal–Wallis test). **d** Correlation analysis of serum biomarkers and differentially distributed species between groups of mice (Kruskal–Wallis test, *P < 0.05. **P < 0.01). **e** LEfSe showing the differences in abundance between groups. **f** Box plot of differences in KEGG pathways between groups predicted by PICRUSt2 (Kruskal–Wallis, *P < 0.05).

To reveal the changes in intestinal microbes in D/L-induced ALF mice and the potential regulatory role of *Ep300*i, we collected mouse ileal contents and performed 16S rRNA-seq. Alpha diversity analysis showed that *Ep300*i reduced the abundance of intestinal microorganisms in mice, and the Simpson and Shannon indices were reduced in the D/L+*Ep300*i group (Fig. 7a). Changes in the overall structure of the gut microbiota were analyzed using the unsupervised multivariate statistical method UniFrac distance-based principal coordinate analysis (PCoA). PCoA scores showed significant separation between the microbiota of the control group and those of the other two groups (*P* = 0.001), implying that *Ep300*i significantly altered the intestinal microbiota of ALF mice (Fig. 7b).

Then, we found that the relative abundance of five genera, including *Algoriphagus*, *Alloprevotella*, *[Eubacterium]_coprostanoligenes_group*, *NS9_marine_group*, and *Akkermansia*, was increased in the D/L group but was suppressed by *Ep300*i treatment (Fig. 7c). Furthermore, the above five microorganisms were positively correlated with ALT and AST levels; *Algoriphagus* and *NS9_marine_group* were positively correlated with serum iFABP and LPS levels (Fig. 7d). Linear discriminant analysis effect size (LEfSe) analysis also supported the notion that the five microorganisms mentioned above could serve as biomarkers of intestinal changes in ALF mice (Fig. 7e). In previous reports, *Alloprevotella* has been positively associated with high-fructose diet-induced liver injury in mice, *[Eubacterium]_coprostanoligenes_group* has been positively associated with high-fat diet-induced nonalcoholic steatohepatitis-related intestinal ecological dysregulation, and *Akkermansia* has been found to ameliorate APAP-induced liver injury^63,64^. The association of *Algoriphagus* and *NS9_marine_group* with ALF is reported here for the first time. Finally, based on PICRUSt2, we predicted the KEGG pathways of microbial genes in different experimental groups. Interestingly, in ALF mice, the pathways associated with energy metabolism, cell death, transport, and viral infection were upregulated, but the upregulation of these pathways was suppressed by *Ep300*i (Fig. 7f). Surprisingly, consistent with our scRNA-seq results, abnormal energy metabolism, microbial infection and cell death are the main changes in IECs that are affected by ALF, which indicates that there is a strong correlation between the changes in the intestinal microbiome and IEC injury.

### The transcriptional activation of *Ep300* is universal in different ALF models

After comprehensively examining the *Ep300* target genes in the SCENIC database and the most significantly upregulated ALF_DEGs in all cells, we identified six of them that were most significantly upregulated in the intestines of D/L mice. *Celsr3*, a key gene in the development of the mouse enteric nervous system^65^, has previously been shown to be highly expressed in patients with congenital megacolon and to promote dysregulation of the innervation pattern of the intestine^54^. *Gins3* is involved in the formation of replication forks and has been shown to be associated with a variety of epithelial cell carcinogenesis processes^66^. *Sh3bp5* acts as a target of JNK and has been found to maintain its activation by promoting the production of reactive oxygen species in an acute liver injury model^67,68^. *Anxa9*, a calcium-dependent phospholipid-binding protein, is associated with immune infiltration and poor prognosis in cancer tissue^68^. *Plekhm1*, a multivalent endocytic adapter, mediates lysosomal fusion of the autophagic pathway^69^. These *Ep300* target genes are closely associated with intestinal cell autophagy, apoptosis, microbial infection and carcinogenesis, and they can be considered markers of *Ep300* activation in ALF intestinal injury.

**Fig. 8:**
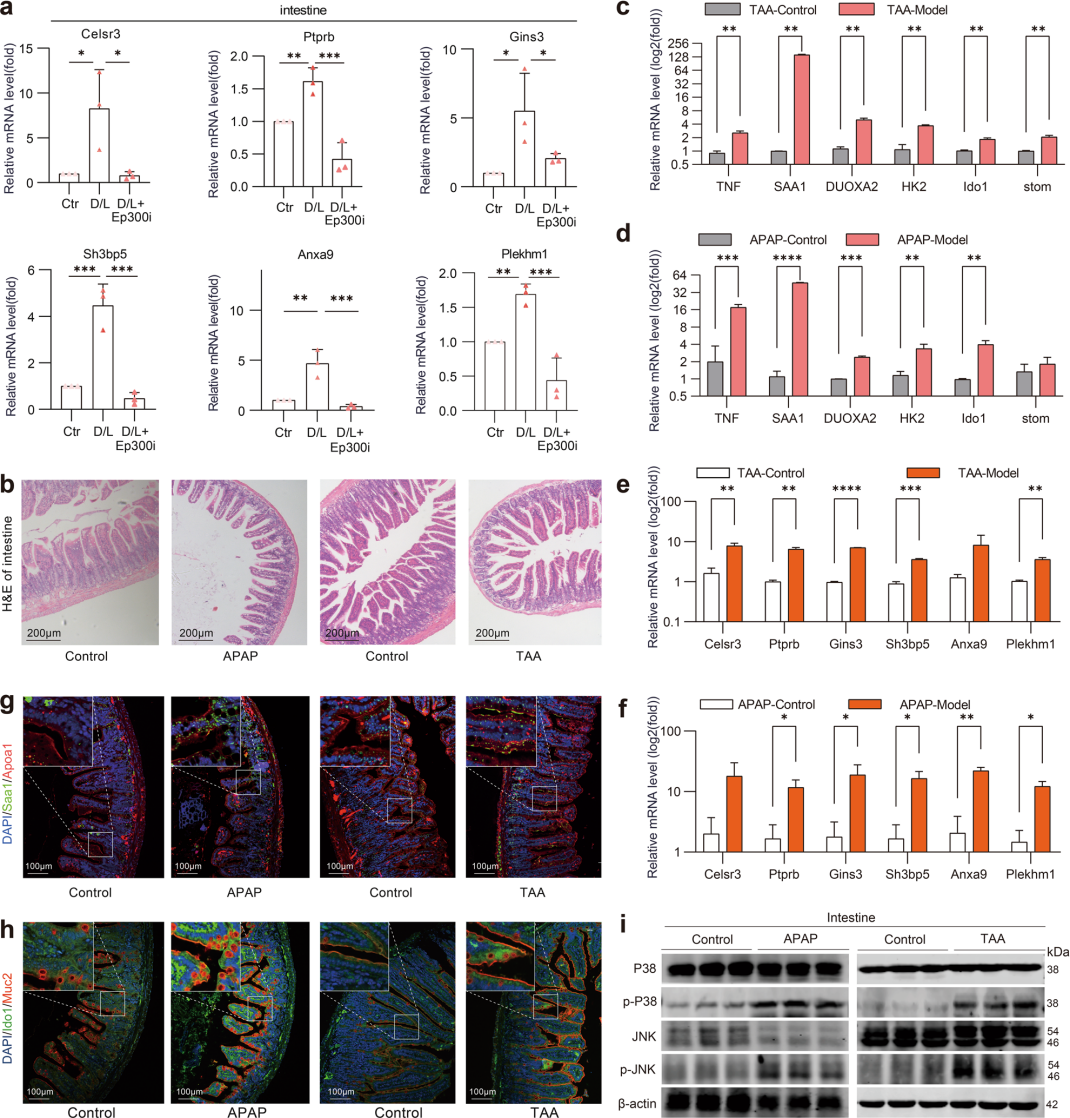
The *Ep300*-dependent transcriptional program is universal in different mouse ALF models. **a** q-PCR analysis of mRNA levels in mouse intestinal. **b** Representative images of H&E staining of mouse intestinal sections. Scale bar, 200 μm. **c**–**f** q-PCR analysis of mRNA levels in mouse intestinal. **g** Representative IF images of *Apoa1* and *Saa1* in mouse ileum. Scale bars, 100 μm. **h** Representative IF images of *Muc2* and *Ido1* in mouse ileum. Scale bars, 100 μm. **i** P38, p-P38, JNK and p-JNK expression in mouse intestinal as analyzed by western blotting assay.

Next, quantitative PCR (q-PCR) was used to measure the expression of the above genes. The results showed that the D/L-induced *Ep300* activation in the intestines of mice was accompanied by upregulation of these target genes, which was significantly inhibited by *Ep300*i (Fig. 8a). To further confirm the universality of *Ep300* activation in ALF, we established ALF models induced by acetaminophen (APAP) or thioacetamide (TAA) and conducted relevant verification. As shown in Fig. 8b, intestinal villus vacuolation increased significantly and was accompanied by villus destruction and disorder in mice of both the APAP- and TAA-induced models. We also found that key ALF_DEGs of scRNA-seq in the intestine of D/L-induced ALF mice (Fig. 8c, d) and *Ep300* target genes (Fig. 8e, f) were significantly upregulated in the intestines of APAP- or TAA-induced ALF mice. Further, the numbers of *Saa1*^+^*Apoa1*^+^ enterocytes and *Ido1*^+^*Muc2*^+^ goblet cells (Fig. 8g, h), and the phosphorylation of P38 and JNK (Fig. 8i) were increased in the intestines of APAP- or TAA-induced ALF mice (Fig. 8i). These data confirm the universality of *Ep300* transcriptional activation in mice with ALF and suggest that *Ep300* is a therapeutic target to protect the intestines of ALF mice and relieve ALF.

**Fig. 9:**
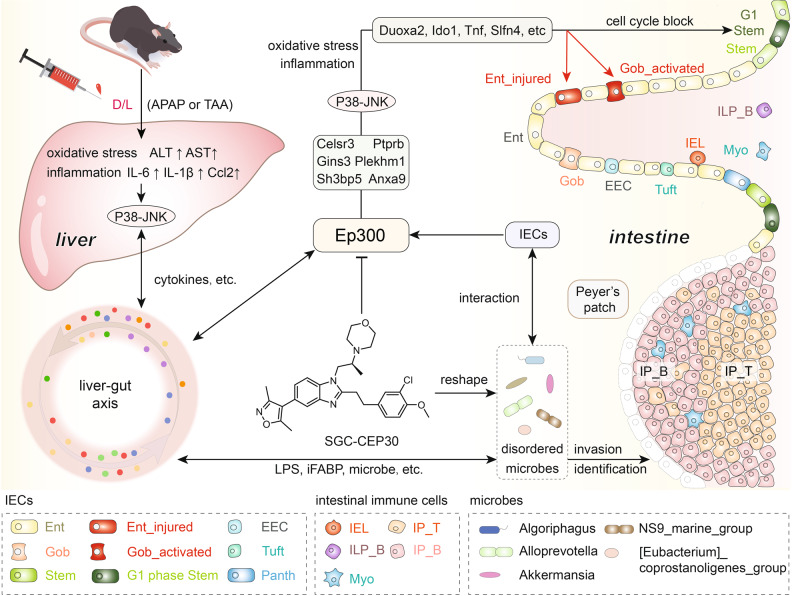
Summary diagram of intestinal cell heterogeneity and the potential mechanism of *Ep300*i in ALF mice. Ent enterocytes, Gob goblet cells, IEL intraepithelial lymphocytes, IP_T intestinal PPs T cells, ILP_B intestinal lamina propria B cells, IP_B intestinal PPs B cells, Myo myeloid cells.

Upon analyzing the results obtained with the highly representative D/L-induced ALF model, we speculated that *Ep300*i alleviates ALF through the following mechanism: *Ep300*i restrains inflammation and oxidative stress in the dysregulated cluster of IECs through the P38-JNK pathway and also regulates the gut microbial composition and metabolism, correcting intestinal dysbiosis and thereby protecting IECs and attenuating ALF (Fig. 9).

## Discussion

With the development of technologies, the critical roles of the gut–liver axis in liver immunity, metabolism, detoxification and other processes have been gradually revealed^61^. The influence of the gut–liver axis and its relationship to microbes have been reported in ALF^5^ and acute-on-chronic liver failure^64^. However, existing studies have only focused on the liver and intestinal microbiome; they have ignored the intestinal part of the gut–liver axis, including changes in the transcriptomes of various intestinal cells, related signaling transduction pathway and cell communication in the process of ALF. To reveal changes in the intestine in ALF mice, we performed scRNA-seq on IE and PPs without IE. We comprehensively characterized the cellular landscape and gene expression changes within the IE and PPs in ALF mice, proposed *Ep300* as a possible target for treating ALF and performed exhaustive validation in three mouse ALF models.

The pathogenesis of ALF that is induced by different chemicals or drugs is slightly different, but there are some commonalities. The current consensus is that the metabolic imbalance and excessive accumulation of toxins and their metabolites lead to oxidative stress, mitochondrial damage and the inflammatory response, which further activates multiple signaling pathways and promotes death of liver and intestinal cells^70^. In D/L-induced ALF, D-GALN is the hepatotoxin that targets hepatocytes, and LPS, as a bacterial product, accelerates the process of liver injury leading to ALF. ALF is usually accompanied by a systemic cytokine storm, with ultra-high levels of cytokines in the circulatory system inducing damage to the intestinal mucosa and disruption of mucosal immunity in the gut, leading to changes in gut microbiology. The disturbed gut ecology leads to a disruption of the intestinal barrier, resulting in more toxic substances (e.g., bacteria and microbial products) “leaking” from the IE into the circulation; this further induces an imbalance in the gut–liver axis and thus accelerates the progression of ALF. In summary, D/L-induced ALF mice represented the characteristics of ALF to the greatest extent, especially the gut–liver axis-related characteristics; our scRNA-seq data of this model will lay a foundation for the study of the ALF-related gut–liver axis.

The IE is a key component of the intestinal barrier that plays a critical role in the gut–liver axis homeostasis. Damage to the IE causes a leaky gut, leading to the invasion of intestinal microbes and the release of microbial products into the circulation, which subsequently triggers the progression of liver disease. In ALF mice, as reported in many liver diseases, disordered cytokines and gut microbes in the gut–liver axis, among other factors, contribute to the deterioration of liver disease by causing intestinal damage. Therefore, targeting IECs to improve liver disease through the gut–liver axis has great potential^3,8^. Based on our scRNA-seq data, we precisely delineated the makeup of the IE and identified a cluster of injured enterocytes. Moreover, ALF induced a cycle block in intestinal stem cells and activated the specific goblet cell cluster Gob1. The critical role of the IE in the gut–liver axis underscores the significance of our work, but the critical signaling molecules for hepatic and intestinal signaling in ALF still need to be elucidated. Consistent with D/L-induced ALF, APAP-induced ALF, the most prevalent ALF representative model, is also associated with severe IECs damage^64^. Herein, in addition to using the D/L model, we validated the activation of *Ep300* in the intestines of APAP- and TAA-induced ALF mice, identifying these three mouse ALF models with similar patterns of transcriptional activation in the intestine. The limitation of this study was the lack of related data on clinical samples, which will be addressed in our further research.

SGC-CBP30, a potent inhibitor of the highly selective CBP/p300 bromodomain, has shown therapeutic potential in a variety of diseases, including cancers, gastrointestinal syndromes, sepsis, and organ fibrosis^62,71–73^. We investigated the pharmacological effects of SGC-CBP30 in ALF for the first time and confirmed that pharmacological inhibition of *Ep300* could effectively inhibit D/L-induced ALF in mice and ameliorate liver injury, coagulation abnormalities, and inflammation. In comparison to that in SPF mice, the hepatoprotection of SGC-CBP30 was diminished in ABX mice and its intestinal protection was lost, suggesting that its intestinal protective mechanism requires the involvement of intestinal microbes. Based on the results of 16S rRNA-seq, we found that SGC-CBP30 regulates the specific flora associated with intestinal and liver injury and inhibits energy metabolism/cell death and other pathways of intestinal microorganisms, thus protecting IECs. Extensive research on gut microbes has focused on the removal of gut microbes or the inhibition of Toll-like receptors associated with microbial recognition^5,74,75^. Although antibiotic treatment attenuates D/L-induced ALF, nonselective depletion of gut microbes simultaneously disrupts the microbial balance of the gut–liver axis and leads to damage to the liver and intestine (especially the ileum^76^). Consistent with this, our data showed that in ABX mice, minor liver and intestinal damage was present prior to D/L treatment, as evidenced by gallbladder enlargement, elevated serum AST and iFABP levels and intestinal villus damage, implying that nonselective gut microbiota depletion may have only a limited benefit/risk ratio. Therefore, selective modulation of the gut microbes is superior to nonselective removal of microbes. However, fecal transplantation, a highly respected strategy for selective modulation of the gut flora, has a slow onset of action that conflicts with the rapid progression of ALF. Therefore, we believe that an initial intent based on IEC protection, rather than microbial correction, during the progression of liver failure may result in better benefits from ALF treatment. Aside from *Ep300*, we also identified other potential targets for the treatment of ALF, such as *Zbtb7b* and Serum response factor (*Srf*). *Zbtb7b* is an essential gene for enterovirus clearance and is critical for the viral control function of the IEL^32^. *Srf* is a TF that mediates plasticity in smooth muscle cells and inhibits apoptosis of intestinal smooth muscle cells^77^. Both of these genes are potential therapeutic targets for ALF and warrant further investigation in the future.

Taken together, this study described the intestinal panorama of ALF mice in detail, clarified the root causes of intestinal cell-related abnormalities in ALF, and provided a basis for ALF research and drug development based on the gut–liver axis. Using *Ep300*i alone or in combination with other drugs to treat ALF is worthy of attention in the future.

## Materials and methods

### Preparation of single-cell suspensions

Single cells from the crypt-associated IE were isolated by a modified method from the previously reported method^38^. Briefly, the ileum with PPs removed was cut longitudinally and washed three times with ice-cold PBS until the supernatant was clarified. The intestine was cut into pieces ~2 mm in length, placed in 10 mM EDTA-PBS, incubated upside down at 4 °C for 20 min, and then shaken vigorously for 5 min, and the supernatant was collected. The incubation was repeated twice, and all supernatants were combined and centrifuged at 20× *g* for 4 min. The precipitate (enriched for crypts) was washed with ice-cold PBS and centrifuged at 350× *g* for 3 min. The precipitate was resuspended with TrypLE™ Express (Gibco, Cat: 12604039) supplemented with 5 mg/mL dispase (Roche), and the cells were observed to dissociate in a water bath at 37 °C for 20 min. When the cells dissociated into a large number of single cells, a large amount of ice-cold PBS was added to terminate the digestion. The single-cell suspension was then passed through a 40-μm filter. The Dead Cell Removal Kit from Miltenyi biotec (Cat: 130-090-101) was used to remove dead cells before scRNA-seq.

Single cells from PPs cells were isolated by a modified method from the previously reported method^78^: PPs were collected and incubated in Hank’s balanced salt solution (HBSS) supplemented with 5 mM EDTA, 1 mM DTT, and 20 μM HEPES at pH 7.2 for 20 min at 37 °C followed by centrifugation at 20× *g* for 5 min to remove the supernatant (to remove IE-associated cells). The precipitate was then chopped and dissociated by incubation in HBSS containing dispase (5 mg/mL, Roche, Cat: 04942078001), collagenase D (0.5 mg/mL, Biofox, Cat: 2091), and DNaseA (0.5 mg/mL, Roche, Cat: 10104159001) for 20 min at 37 °C. The single-cell suspension was then passed through a 40-μm filter. Cells were washed with cold PBS and then suspended in PBS containing 1% BSA before scRNA-seq.

### ScRNA-seq

The scRNA-seq data were processed and quantified as below: First, the mm10 reference used to align the reads was obtained from 10× Genomics. Then, the sequenced FASTQ files were compared to the refdata-cellranger-mm10-3.0.0 mouse reference genome using STAR software. Finally, the BD genomics rhapsody (1.9.1) module was used to generate a matrix of feature and cell barcodes.

### Quality control, clustering, marker gene identification

Normalization, clustering, differential gene expression analysis, and visualization were performed using the R package Seurat (4.2). The parameters used for cell quality control were 6000 > nFeature_RNA > 200, 40000 > nCount_RNA > 500, log_10_GenesPerUMI > 0.80, percent.mt < 20, percent.rp < 20. Cells were clustered using the cluster identification method implemented in the FindClusters function and visualized using the RunUMAP or RunTSNE function. The FindMakers function was used to identify specific marker genes for cell clusters. The strategy for all marker gene screening in this study was to average expression relative to all other clusters, with Bonferroni-corrected *P* values of less than 0.05 for average expression of marker genes in the target cluster and a minimum log2 fold change of 0.25. Classical marker genes and the top differentially expressed genes were used to annotate the cell type of each cluster.

### Cell cycle analysis

We use the “cyclone” function of the Scran package (1.24.1) to identify specific changes in cell cycle phases in different intestinal stem cell clusters. The “cyclone” function scores each cell by calculating the similarity of the characteristics of the target cell population to the training cell set based on the set of cell cycle-related “maker gene pairs” generated from the training set of cells. The “cyclone” function will assign a predicted cell cycle to each cell based on its score.

### Key visualization information

R package ggplot2 (3.3.6) was used for data visualization; marker gene average expression heatmap was generated by the R package scRNAtoolVis (0.0.4) and volcano map was drawn by R package Enhanced Volcano (1.14.0). Graphpad (8.0.2) was also used in small amounts for data visualization, and some vector diagrams used for schematic purposes are obtained from Freepik (https://www.freepik.com/).

### GO and KEGG analysis

GO enrichment analysis (*P* value adjusted by Bonferroni correction < 0.01) or KEGG enrichment analysis using the R package clusterProfiler for marker genes in each cluster (functions compare Cluster, enrichGO, and enrichKEGG functions) or differential genes in different groupings^77^.

### Single-cell trajectory analysis

Single-cell trajectories were constructed using Monocle (2.0) based on the results obtained with Seurat (4.2). Genes included in the analysis were selected by applying the following criteria: expressed in more than 10 cells, and mean expression level greater than 0.1. The top 2000 genes with the highest significance were selected as trajectory-defining genes, and then the data were downscaled using the reverse graph embedding (DDRTree) algorithm. Differential-Gene-Test function was used to find differential genes at the pseudotime.

### Cell–cell communication analysis

CellChat^79^, a tool capable of quantitatively inferring and analyzing intercellular communication networks from scRNA-seq data was applied. CellChat results were used to reveal the afferent communication patterns of target cells and the efferent communication patterns of secretory cells and to compare the signaling differences between control and model groups. Further, we compared the correspondence of the differential pathways with different cell subpopulations.

### TF regulatory analysis and protein–protein interaction networks

SCENIC is a computational method for the simultaneous reconstruction of gene regulatory networks and cell state identification from scRNA-seq data (http://scenic.aertslab.org)^80^. SCENIC analysis was performed on specific populations of cells included in the analysis and regulators were calculated based on TFs or their target genes, and TFs with significantly upregulated activity were involved in further analysis.

In enterocytes, the strategy used to screen for TFs with greater than mean or median activity in Ent5 and less than mean or median TF activity in immature Ent1 to Ent3. In goblet cells, EECs, Panth cells and Tuft cells, based on the higher degree of activation in goblet cells, we screened for TFs with greater than mean or median activity TFs and screened for TFs with less than mean or median activity in the less activated goblet cells (Gob2 and Gob3). We constructed protein–protein interaction networks for TFs activated in Ent5 or Gob1 using String version 11.5 (https://cn.string-db.org/)^81^ and used the CytoNCA plugin of Cytoscape software (3.9.1) Betweenness algorithm to assess protein interaction network centrality^82^.

### Animal experiment protocol

Eight-week-old male C57BL/6 mice weighing 22–24 g were obtained from Vital River Laboratory Animal Technology Co. Ltd (Certificate SCXK2021-0006; Beijing, China). All animals were raised in a room with a temperature of 24 ± 1 °C and a 12-h light-dark cycle for 7 days before the experiment. The current study was conducted by the International Guiding Principles for Biomedical Research Involving Animals, which has been approved by the Institutional Animal Care and Use Committee, Huazhong University of Science and Technology (2021 IACUC Number: 3080). To induce ALF by D/L (D-GALN, 500 mg/kg, Sigma-Aldrich, Cat: G1639; Lipopolysaccharides, 5 μg/kg, Sigma-Aldrich, Cat: L2630; both dissolved in PBS) and test the hepatoprotective and enteroprotective effect of *Ep300* inhibitor SGC-CEP30 (MedChemExpress, Cat: HY-15826), mice were randomly divided into three groups (*n* = 6–8): Control group (PBS + Corn oil), D/L group (D/L + Corn oil), D/L + *Ep300*i group (D/L + SGC-CEP30). SGC-CEP30 dissolved in 10% DMSO and 90% Corn oil, and administered intraperitoneally (50 mg/kg) 1 h before D/L treatment. After the D/L challenge for 4.5 h, the liver, intestine and blood were collected for further experiments. The blood was centrifuged at 3000× *g* for 20 min at 4 °C to harvest mouse serum. To induce ALF with APAP and TAA, mice were randomly divided into four groups (*n* = 4): APAP control group, APAP model group (APAP, 500 mg/kg dissolved in PBS, Aladdin, Cat: A105808), TAA control group and TAA model group (TAA, 300 mg/kg dissolved in PBS, Aladdin, Cat: T118452). After APAP/TAA induced for 23 h, the intestines of mice were collected for further experiments.

### Broad-spectrum antibiotic treatment

ABX treatment was administered according to previously published protocols. Broad-spectrum ABX mix was diluted in sterilized water consisting of Ampicillin (1 g/L, Sigma-Aldrich, Cat: A9518), Neomycin (1 g/L, Sigma-Aldrich, Cat: N1876), Vancomycin (0.5 g/L, Sigma-Aldrich, Cat: 94747), and Metronidazole (1 g/L, Sigma-Aldrich, Cat: M3761). ABX solution was used as the only drinking water source for mice for 15 days (the consumption was checked and topped up every 2 days). To assess the effects of SGC-CEP30, ABX-treated mice were randomly divided into three groups (*n* = 6–8): ABX group (ABX + PBS + Corn oil), ABX + D/L group (ABX + D/L + Corn oil), ABX + D/L + *Ep300*i group (ABX + D/L + SGC-CEP). After the D/L challenge for 4.5 h, the liver and blood were collected for further experiments. The blood was centrifuged at 3500× *g* for 10 min at 4 °C to harvest serum.

### ELISA assays

Commercially available ELISA kits, including Mouse intestinal fatty-acid binding protein (iFABP) ELISA KIT and Mouse LPS ELISA KIT, were used to assess serum marker levels following the manufacturers’ instructions (Nanjing Boyan Biotechnology Co., Ltd). Mouse serum was diluted 1:10 to measure iFABP and LPS levels.

### RNA isolation and qPCR

The total RNA of mice liver was isolated with TRIzol™ Reagent (Thermofisher, Cat: 15596026) and cDNA was synthesized by HiScript® II Q RT SuperMix for q-PCR (Vazyme, Cat: R223-01). SYBR Green Mix was purchased from Biosharp (Biosharp, Cat: BL698A). The primers used are listed in Supplementary Table S2.

### Measurement of ALT and AST levels

The levels of ALT and AST in mouse serum were measured by the Alanine aminotransferase Assay Kit and Aspartate aminotransferase Assay Kit (Nanjing Jiangcheng, Cat: C009-2-1 & C010-2-1).

### Western blotting assay

We thawed the tissue and used the appropriate RIPA buffer (Beyotime, Cat: P0013B) to fully homogenize. The sample was lysed at 4 °C for 20 min, then 12,000× *g* centrifuged at 4 °C for 15 min, and the supernatant was collected and the protein concentration was determined by a BCA protein assay kit (Beyotime, Cat: P0012S). Then, we denatured the protein samples at 95 °C for 10 min after mixed with the loading buffer. Samples of 10–30 μg protein were separated by 10%–15% SDS-polyacrylamide gel and then electrophoretically transferred to a nitrocellulose filter membrane (Pall Corporation, Cat: 66485) with 20% methanol. The membrane was blocked with (5% (w/v) nonfat dry milk in TBS) for 1 h and then incubated overnight at 4 °C with specific primary antibodies (diluted according to manufacturer’s instructions, ranging from 1:200 to 1:100,000). These membranes were then washed with TBST buffer solution three times and incubated for 1 h with DyLight™ 800 or DyLight™ 600-conjugated secondary antibody (1: 15,000). Lastly, the membranes were visualized by the Odyssey® CLx Imaging System (LI-COR). Antibodies purchased from Cell Signaling Technology include SAPK/JNK (Cat: 9252), Phospho-SAPK/JNK (Thr183/Tyr185) (Cat: 4668), p38 MAPK (Cat: 8690), Phospho-p38 MAPK (Thr180/Tyr182) (Cat: 4511), Anti-mouse IgG (H + L) (DyLight™ 800 4X PEG Conjugate) (Cat: 5257), Anti-rabbit IgG (H + L) (DyLight™ 680 Conjugate) (Cat: 5366), and from Proteintech β-actin(Cat: 66009-1-Ig).

### TUNEL staining

TUNEL staining was performed with In Situ Cell Death Detection Kit (Roche, Cat: 11684817910) according to the manufacturer’s instructions. In brief, paraffin sections were prepared and treated with proteinase K working solution at 37 °C for 25 min to repair the antigen. After washing with PBS, the permeabilization solution was added at room temperature, after 20 min washed with PBS. Then, TDT and dUTP were mixed at a ratio of 1:9, covered the tissue, and incubated at 37 °C for 2 h. Nikon AX/AX R Confocal Microscope System was used for imaging.

### IF

Paraffin sections were prepared and blocked with 3% BSA for 30 min. Sections were incubated overnight at 4 °C with the following primary antibodies: Cd3d (1:200, proteintech, 16669-1-AP), Gzmb (1:200, proteintech, Cat: 13588-1-AP); Cd79b (1:300, proteintech, Cat: 21063-1-AP); Ido1 (1:200, proteintech, Cat: 13268-1-AP); Muc2 (1:200, proteintech, Cat: 27675-1-AP); Apoa1 (1:200, proteintech, 14427-1-AP); Saa1 (1:200, ABclonal, Cat: A14553). On the second day, the section was incubated with Cy3/488-labeled secondary antibody (1:200–1:500, Abcam, Cat: ab6939, ab150077 or ab97035) for 50 min at room temperature. DAPI staining solution (Solarbio, Cat: C0065) was used to stain the nucleus at room temperature for 10 min and then the sections were blocked with an anti-fluorescence quenching blocking agent (Solarbio, Cat: S2110). Nikon AX/AX R Confocal Microscope System was used for imaging.

### Hematoxylin-eosin (H&E) staining and IHC

Tissues were fixed in 4% paraformaldehyde, embedded in paraffin, and then cut into 5-μm-thick sections. These sections were stained with H&E to evaluate the histopathological injury. For IHC, sections were blocked with 3% BSA or 10% rabbit serum prepared in PBS for 30 min at room temperature and then incubated overnight at 4 °C with ZO-1 antibody (1:200, Santa Cruz, Cat: sc-33725). On the second day, the section was incubated with secondary antibody for 50 min at room temperature, and freshly prepared DAB chromogenic solution was added dropwise. The nucleus was hematoxylin counterstained and dehydrated, and the sample was mounted with neutral gum. The Olympus CKX53 microscope was used for imaging.

### 16S-targeted bacterial composition profiling

Small intestine contents were collected postmortem, flash-frozen in liquid nitrogen, and stored at −80° C. DNA was extracted from the samples with MagPure Soil DNA LQ Kit (Magen, Cat: D6356-02) according to the manufacturer’s protocol. The V4 fragment of the 16S rRNA gene was amplified using forward primer TACGGRAGGCAGCAG and reverse primer AGGGTATCTAATCCT. PCR was performed using Takara Tks Gflex DNA Polymerase (R060B) with 50 ng of genomic DNA input, and 0.4 μM of each primer in 30 μL reactions. PCR amplification, or its absence in negative controls, was verified by agarose gel electrophoresis. Samples were pooled equimolarly with Qubit dsDNA Assay Kit (Life Technologies, Cat: Q32854). QIIME2 (2020.11) and PICRUSt2 (2.3.0b0) were used for further data analysis.

## Supplementary information

Supplementary Figures

Supplementary Table S1

Supplementary Table S2

## Data Availability

The scRNA-seq data (GSA: CRA011247) and 16S rRNA-seq data (CRA011259) generated in this study were deposited in the Genome Sequence Archive (GSA) database at the National Genomics Data Center (NGDC, https://bigd.big.ac.cn/).
