# Supplementary material for: Single-cell transcriptomics reveals intestinal cell heterogeneity and identifies Ep300 as a potential therapeutic target in mice with acute liver failure
Source: Cell Discov. 2023 Jul 25;9:77. doi: 10.1038/s41421-023-00578-4 (PMC10366100; doi:10.1038/s41421-023-00578-4)
Supplement: Supplementary file 1 — Supplementary Figures [file 41421_2023_578_MOESM1_ESM.pdf]

Supplementary Fig. S1

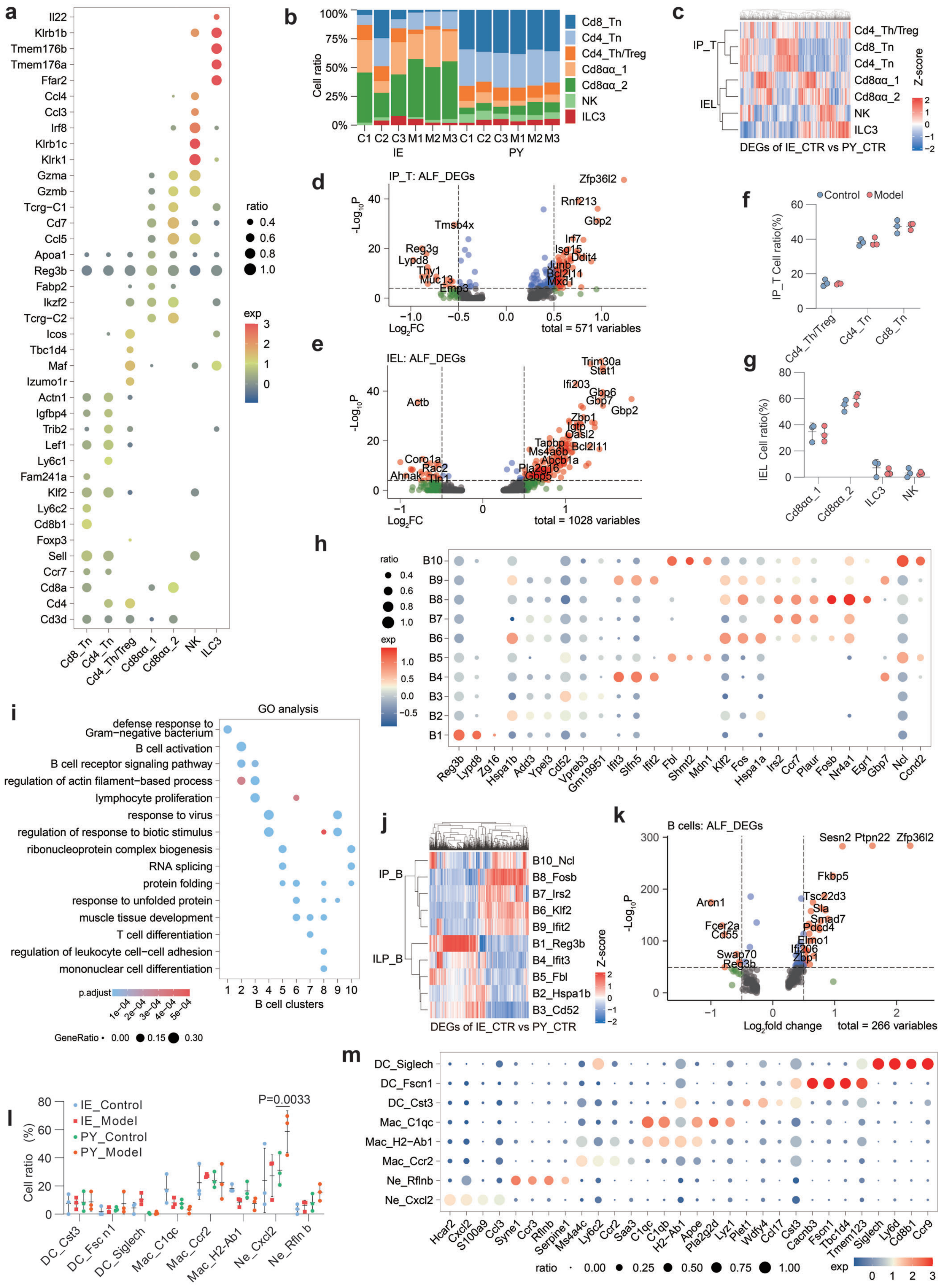

Supplementary Fig. S2

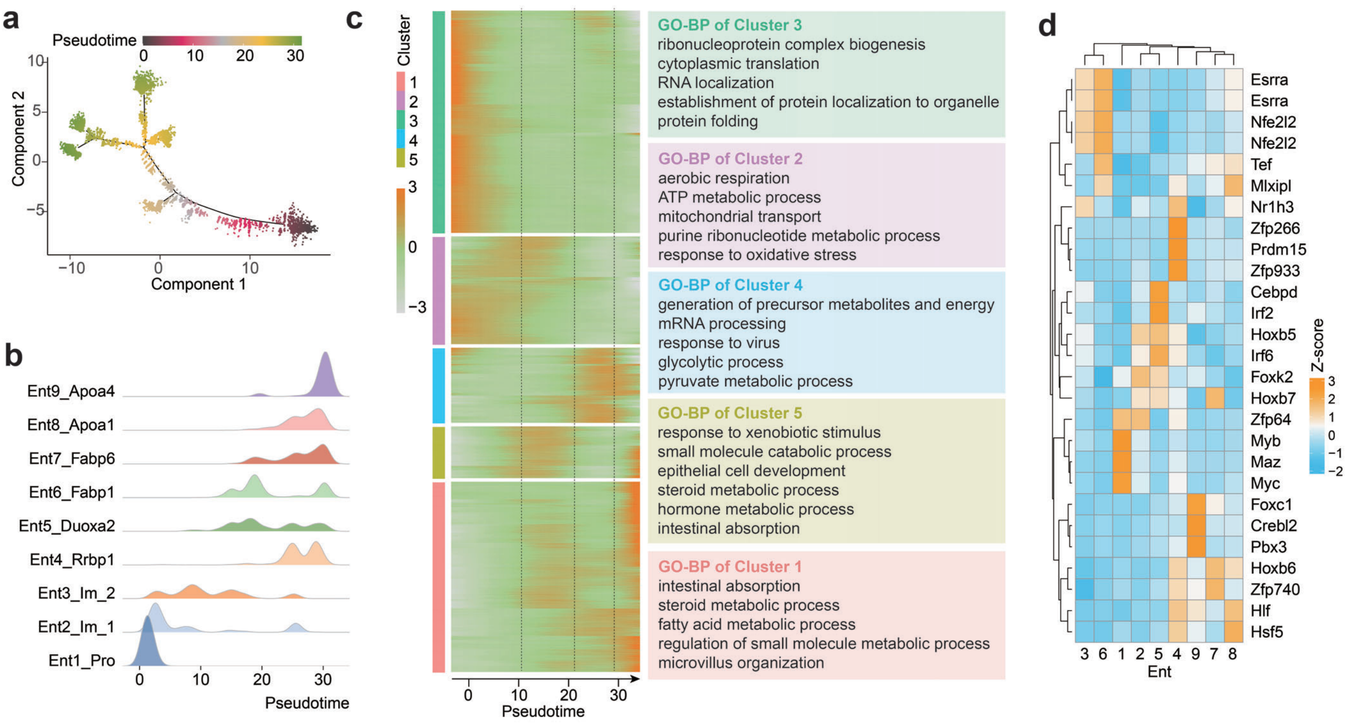

Supplementary Fig. S3

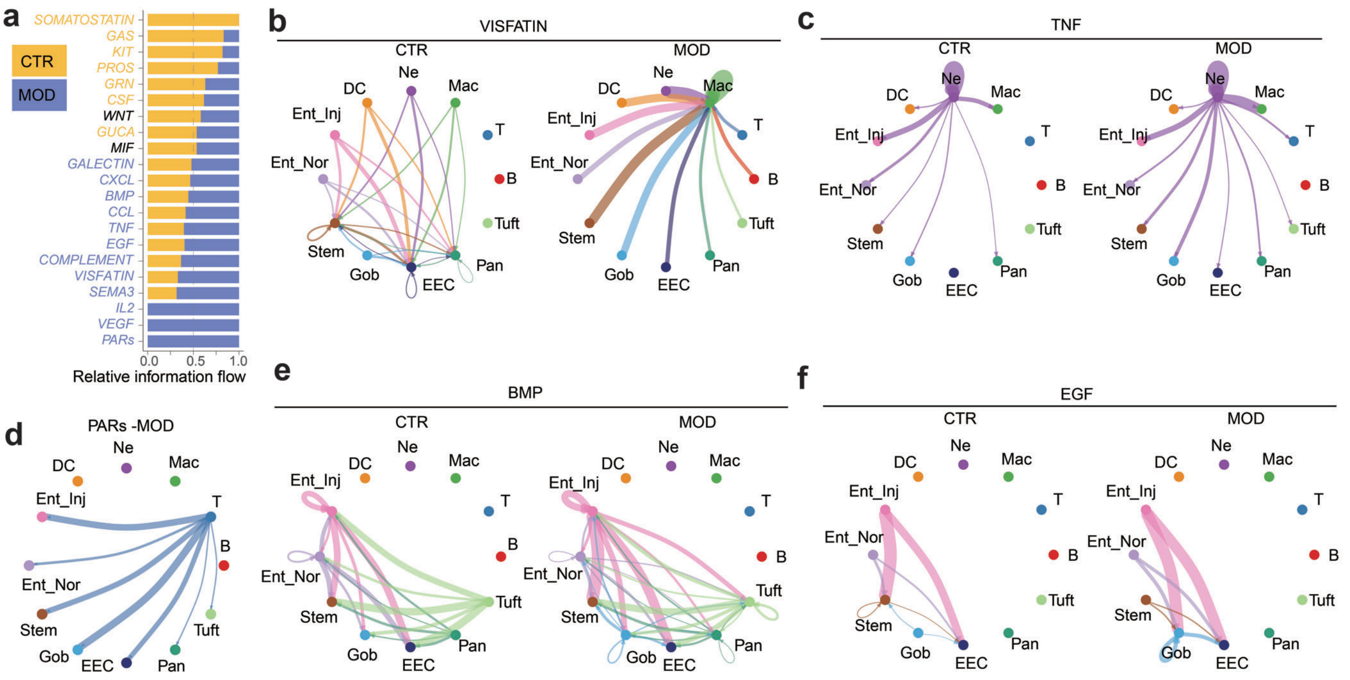

Supplementary Fig. S4

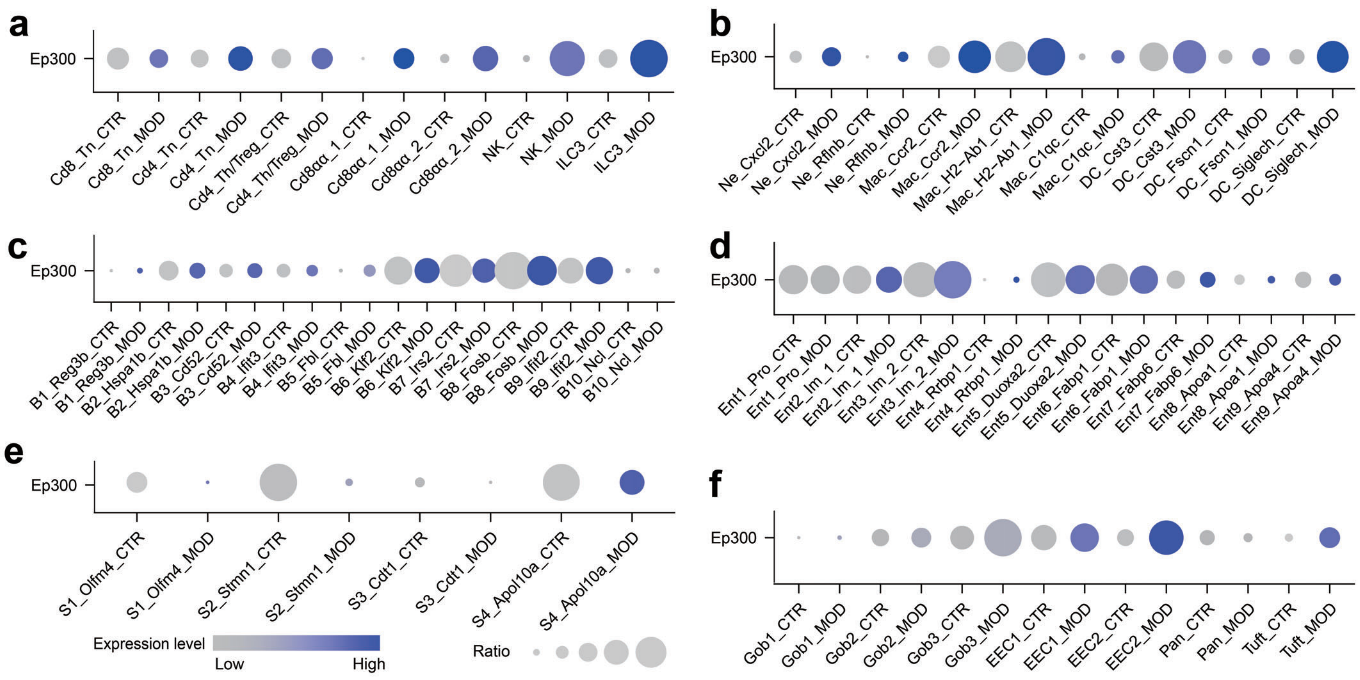

Supplementary Fig. S1 Heterogeneity of intestinal immune cells in D/L-induced ALF mice.

a Dot plot depicting expression of the top 5 marker genes of T/NK cells. Expression values are normalized and scaled averages. **b** Proportion plot of all T cell clusters in intestine. **c** Clustered heatmap of tissue differential gene average expression. **d** Volcano plot of ALF_DEGs in IP_T. **e** Volcano plot of ALF_DEGs in IEL. **f** Proportion plot of intestinal Peyer's patch T cell clusters. Mean ± SD, two-way ANOVA, n = 6 mice. **g** Proportion plot of IEL cell clusters. Mean ± SD, two-way ANOVA, n = 6 mice. **h** Dot plot of gene expression for each B cell cluster top 3 marker genes. **i** GO analysis results of B cell cluster's marker genes. **j** Clustered heatmap of tissue_DEGs average expression in B cells. **k** Volcano plot of ALF_DEGs in B cells. **l** Proportion plot of myeloid cells. Mean ± SD, two-way ANOVA, n = 6 mice. **m** Dot plot of gene expression for each cell cluster top 5 marker genes in myeloid cells.

Supplementary Fig. S2 Pseudotime analysis and transcription factor analysis of enterocytes.

a Pseudotime axis of enterocytes. **b** Pseudotime peak plots for enterocyte clusters. **c** Clustering heatmap and GO enrichment analysis of pseudotime-related gene expression in enterocytes. **d** Heatmap of TFs specifically activated in clusters of enterocytes.

Supplementary Fig. S3 Altered cellular communication in injured enterocytes.

a All significant signalling pathways were ranked based on their differences in overall information flow within the inferred networks between control group and model group. **b-f** The circle plot shows the communication strength of the differential pathways between the different groups.

Supplementary Fig. S4 Ep300 is widely upregulated in the intestinal cells of D/L-induced ALF mice.

a Dot plot of Ep300 expression in T/NK cell clusters in the control and model groups.

b Dot plot of Ep300 expression in myeloid cell clusters in the control and model groups.

c Dot plot of Ep300 expression in B cell clusters in the control and model groups.

d Dot plot of Ep300 expression in enterocyte clusters in the control and model groups.

e Dot plot of Ep300 expression in stem cell clusters in the control and model groups.

f Dot plot of Ep300 expression in specific IEC clusters in the control and model groups.
